# Clinical validation of RCSMS: A rapid and sensitive CRISPR-Cas12a test for the molecular detection of SARS-CoV-2 from saliva

**DOI:** 10.1371/journal.pone.0290466

**Published:** 2024-03-25

**Authors:** Joaquín Abugattas-Núñez del Prado, Angélica Quintana Reyes, Julio Leon, Juan Blume La Torre, Renzo Gutiérrez Loli, Alejandro Pinzón Olejua, Elena Rocío Chamorro Chirinos, Félix Antonio Loza Mauricio, Jorge L. Maguiña, Piere Rodriguez-Aliaga, Edward Málaga-Trillo

**Affiliations:** 1 Facultad de Ciencias y Filosofía, Universidad Peruana Cayetano Heredia, Lima, Perú; 2 IMS RIKEN Center for Integrative Medical Sciences, Japan; 3 University of California San Francisco, San Francisco, California, United States of America; 4 Department of Computer Science, Christian-Albrecht University of Kiel, Kiel, Germany; 5 Hospital Nacional Guillermo Almenara Yrigoyen, EsSalud, Lima, Perú; 6 Hospital Nacional Edgardo Rebagliati Martins, EsSalud, Lima, Perú; 7 Instituto de Evaluación de Tecnologías en Salud e Investigación (IETSI), EsSalud, Lima, Perú; 8 Department of Biology, Stanford University, Stanford, California, United States of America; Imam Abdulrahman Bin Faisal University, SAUDI ARABIA

## Abstract

Peru’s holds the highest COVID death rate per capita worldwide. Key to this outcome is the lack of robust, rapid, and accurate molecular tests to circumvent the elevated costs and logistics of SARS-CoV-2 detection via RT-qPCR. To facilitate massive and timely COVID-19 testing in rural and socioeconomically deprived contexts, we implemented and validated RCSMS, a rapid and sensitive CRISPR-Cas12a test for the molecular detection of SARS-CoV-2 from saliva. RCSMS uses the power of CRISPR-Cas technology and lateral flow strips to easily visualize the presence of SARS-CoV-2 even in laboratories with limited equipment. We show that a low-cost thermochemical treatment with TCEP/EDTA is sufficient to inactivate viral particles and cellular nucleases in saliva, eliminating the need to extract viral RNA with commercial kits, as well as the cumbersome nasopharyngeal swab procedure and the requirement of biosafety level 2 laboratories for molecular analyses. Notably, RCSMS performed outstandingly in a clinical validation done with 352 patients from two hospitals in Lima, detecting as low as 50 viral copies per 10 μl reaction in 40 min, with sensitivity and specificity of 96.5% and 99.0%, respectively, relative to RT-qPCR. The negative and positive predicted values obtained from this field validation indicate that RCSMS can be confidently deployed in both high and low prevalence settings. Like other CRISPR-Cas-based biosensors, RCSMS can be easily reprogrammed for the detection of new SARS-CoV-2 variants. We conclude that RCSMS is a fast, efficient and inexpensive alternative to RT-qPCR for expanding COVID-19 testing capacity in Peru and other low- and middle-income countries with precarious healthcare systems.

## Introduction

After sweeping across Asia and Europe, COVID-19 took main stage in Latin America. In Perú, the impact of the pandemic has been exceptionally grim, totaling over 218,625 confirmed COVID-19 deaths as of late January 2023, which equals to 663.07 deaths per 100K population [1]. It might seem paradoxical that one of the first Latin American countries to implement prolonged lock downs, travel restrictions and other measures would end up producing the highest COVID-19 lethality in the world [1, 2]. However, socioeconomic disparity, outdated healthcare infrastructure and failure to apply science-based approaches like testing, tracing and isolation (TTI) help explain this outcome. In particular, the early decision by health officials to monitor the spread of the pathogen using low-quality serological tests was a key fatal error that aggravated the first pandemic wave and led to the saturation of hospitals nationwide. Sadly, the prolonged health emergency particularly affected vulnerable populations such as indigenous communities, urban and rural areas with low-income, refugees and migrants.

Given the rapid spread of the disease and the arrival of new pandemic waves triggered by novel SARS-CoV-2 variants, it is urgent to implement efficient and scalable testing strategies integrated into a genomic surveillance system to track the appearance and distribution of viral mutations [3]. Obtaining high-quality diagnostic data is essential to correctly monitor the evolution of the pandemic, choose adequate public health strategies and ease the transition to a new normality.

Currently, ~600 COVID-19 diagnostic kits are commercially offered worldwide, designed to detect viral genes and proteins, or anti-viral antibodies generated by infected patients [4]. While antibody tests are recommended for seroprevalence and epidemiological studies, the “gold standard” for viral detection is the quantitative reverse transcription-polymerase chain reaction (RT-qPCR) [5]. Recently, viral antigen detection tests have added speed and accessibility to COVID-19 molecular diagnostics, although they are less sensitive compared to RT-qPCR [6]. In developing countries, RT-qPCR testing at the population level is restricted by poor access to equipment, supplies, and infrastructure produced in the developed world, as well as by its long processing times, the need for trained personnel and high biosafety standards [7]. These limitations have prompted research groups worldwide to develop new alternative, inexpensive and accessible methods for SARS-CoV-2 detection. Moreover, multiplex RT-qPCR methods have been developed for the diagnosis of this virus [8].

DETECTR is a recently developed CRISPR-Cas-based test that detects SARS-CoV-2 RNA extracted from nasopharyngeal swabs with high efficiency [9]. The method consists of two steps: i) a reverse transcription reaction coupled to loop-mediated isothermal amplification (RT–LAMP), which generates multiple DNA copies from viral RNA at a single constant temperature, using nested primers and a DNA polymerase with displacement and replication activities [10] and ii) a CRISPR-Cas12a recognition reaction that targets and detects the RT-LAMP-amplified viral DNA via the indiscriminate, Cas12a-mediated cut of single-stranded DNA reporter molecules that produce either fluorescent or immunochromatographic readouts [9]. Therefore, the Cas12a step can amplify the signal produced in LAMP and facilitate the detection of low viral copy samples, in addition it is more specific to detect LAMP-amplified viral DNA and not LAMP-amplified artifacts. Moreover, this technology requires neither sophisticated equipment nor specialized personnel, providing a low-cost alternative with sensitivity and specificity comparable to RT-qPCR with a processing time of ~40 minutes [6]. The possibility to use lateral flow strips for immunochromatographic detection results in an inexpensive visualization format, similar to a pregnancy test, easy to apply and interpret [9]. In fact, various molecular tests that similarly combine RT-LAMP and CRISPR-Cas have successfully been used to detect Zika, Dengue and HIV viruses in humans, and some coronaviruses in animals [11].

COVID-19 Nucleic Acid Tests, such as DETECTR or RT-qPCR, have two important limitations related to sample collection. On one hand, the nasopharyngeal swab procedure is uncomfortable for patients, risky for sample collection personnel, and it generates the need for swabs and costly viral transport media. On the other hand, the routine extraction of high-quality viral RNA requires expensive commercial kits and longer processing times. Interestingly, recent studies show that saliva samples can be directly lysed and used efficiently for molecular diagnostics without commercial RNA extraction kits, thus eliminating the need for biosafety level 2 facilities and procedures [12–15]. These incubation methods use standard low-cost laboratory equipment and are based on a simple thermochemical reaction that guarantees 1) inactivation of RNases in the sample and 2) lysis of viral particles, thus ensuring the release of viral genetic material and rendering the sample non-infectious [12–15].

In the present study, we report the development, standardization and clinical validation of an adapted procedure that combines the rapid and low-cost thermochemical method for viral RNA preparation with the amplification and detection robustness of DETECTR but using saliva as starting sample instead of nasal swabs. We show that this integrated system -called *Rapid Coronavirus-Sensitive Monitoring from Saliva*, *RCSMS-* successfully detected SARS-CoV-2 RNA under field conditions in patients at two hospitals in Lima, with sensitivity and specificity comparable to DETECTR and RT-qPCR, and a limit of detection of 50 viral copies per 10 μl reaction. Altogether, we show that RCSMS is an efficient detection technology that can be easily implemented at the primary care level, thus providing a tool for the massification of COVID-19 testing in developing countries.

## Materials and methods

### Study design

The present study, aimed at validating the use of RCMS to test for the presence of SARS-CoV-2 in saliva from Peruvian patients, was developed in three stages:

1. *Method optimization*. To establish the appropriate experimental conditions for the molecular detection of SARS-CoV-2 via the RT-LAMP/CRISPR-Cas12a coupled reaction, i.e. DETECTR, synthetic viral RNA was generated from DNA templates and taken as input material (see below), using PCR products as amplification control for DETECTR. This stage evaluated the performance of DETECTR under ideal conditions, in particular: a) the robustness of the RT-LAMP reaction upon changes in reaction time (between 20 and 30 min) and temperature (gradient between 62 and 68°C), b) the limit of detection (LOD) of the CRISPR-Cas reaction (serial dilutions of the RNA substrate for RT-LAMP), c) the robustness of the CRISPR-Cas reaction to changes in the final concentration of Cas12a enzyme and guide RNAs (0.5, 1, 2 and 3X) in the ribonucleoprotein complex (RNP), length of guide RNAs (41 and 44 bp), preincubation time for the formation of the RNP complex (10, 20 and 30 min), length of fluorescent and biotinylated ssDNA probes (5 and 8 bp), final concentration of fluorescent (20, 50 and 100 nM) and biotinylated probes (20, 50, 100, 200, 400, 500 and 600 nM), CRISPR detection time by fluorescence (5–30 min) and immunochromatography (10, 20 and 30 min), amount of RT-LAMP product (1, 2, 3 and 5 μl) and d) the repeatability of measurements (three replicas). These evaluations also allowed us to optimize the efficiency, cost and readout levels of the test, as well as to verify its reproducibility upon variations in instrumentation and operators.
2. *Analytical validation*. To assess the analytical specificity of DETECTR, we relied on *in vitro* data from a similar study [9] and examined the possibility of cross-reactivity in the primers and guide RNAs used here. For this, we ran a comparative *in silico* analysis of the corresponding regions in common human coronavirus sequences (HCoV-HKU1 (NC_006577.2), HCoV-NL63 (NC_005831.2), HCoV-OC43 strain ATCC VR-759 (NC_006213.1), MERS-CoV (NC_019843.3), SARS-CoV (NC_004718) and SARS-CoV-2 (NC_045512). The potential ability of the test to detect viral variants currently circulating in Perú was inferred by aligning the sequences of our DNA primers and gRNAs with the 755 curated viral genomes from Peru deposited on NCBI (https://www.ncbi.nlm.nih.gov/labs/virus/vssi/#/, accessed on May 25, 2023). To evaluate the performance of DETECTR on Peruvian samples under optimal laboratory conditions, we followed the criteria established by the Foundation for Innovative New Diagnostics (FIND, https://www.finddx.org/covid-19/sarscov2-eval/). The retrospective analysis was carried out on 100 anonymized nasopharyngeal swabs with recorded negative (n = 50) and positive (n = 50) results by RT-qPCR. The samples, kindly provided by the Peruvian National Institute of Health (INS), and which lacked medical records, were obtained by the INS during epidemiological evaluations between March and June 2020, and remained stored in their laboratories until they were delivered to our team on June 30, 2020. While these samples were obtained by the INS from patients who signed an informed consent, we did not had direct contact with these patients nor access to these informed consents. Once received at 4°C, the 100 samples were analyzed via DETECTR for E and N viral genes, with two additional (blinded and randomized) repetitions, to determine sensitivity and specificity values, as well as repeatability and limit of detection (analytical sensitivity).
3. Clinical Validation. Since one of the aims of this study aims to establish the use of saliva as initial sample, we firstly evaluated different conditions for the saliva inactivation assay. Specifically, we tested different inactivation variables such as variations in the final concentration TCEP/EDTA (0.5X, 1X, 2X and 5X), temperature (75°C, 80°C and 95°C) and time of inactivation (5, 10 and 15 minutes). The performance of RCSMS under real field conditions was evaluated by means of a prospective cross-sectional, observational study of diagnostic test precision. Saliva and nasopharyngeal samples from 352 study subjects at Guillermo Almenara and Edgardo Rebagliati National Hospitals were tested following a consecutive non-probabilistic sampling scheme. The sample size was determined using the sensitivity and specificity estimation formula in the PASS 11.0 program (NCSS, LLC. Kaysville, Utah, USA), considering an expected sensitivity and specificity of 96% and 99%, respectively, as reported by Mayuramart et al. [16], and assuming a confidence level of 95% and a precision of 5%. Given the lack of information about the positivity rate in these hospitals at the time, equal proportions (50%/50%) were assumed for the presence or absence of SARS-CoV-2 infection in these patients, as suggested by Macfarlane [17]. Regarding eligibility criteria, we selected ambulatory patients over 18 years of age who visited the hospital for SARS-CoV-2 detection having reported clinical symptomatology defined as: a) acute respiratory infection: cough and/or sore throat, general malaise, fever, headache, nasal congestion, shortness of breath, loss of smell, loss of taste, or b) severe acute respiratory infection: temperature ≥ 38°C and cough, with onset within the last 10 days. Subjects who had participated in vaccine clinical trials of vaccination programs against SARS-CoV-2 were excluded from the study, as were subjects with xerostomia, users of antiparkinsonian drugs, antipsychotics, and/or neuroleptics, and patients with more than three weeks of illness counted since the onset of symptoms. Likewise, saliva samples of poor quality (containing blood, food residues or phlegm) and volumes outside the optimal range (1–2 ml) were excluded. Upon being instructed about the study, the patients read and signed the informed consent after which nasopharyngeal swabs and saliva samples were collected following WHO recommendations between 26^th^ of January of 2021 and 4^th^ of September of 2021, and molecularly analyzed in parallel (see below) the same day of collection. For each participant, an absolute code was assigned to the data collection form and the samples, to protect their personal data and maintain the confidentiality of the results. Saliva samples were transferred the same day of the collection at 4°C to our laboratory at UPCH for RCSMS analysis of the E gene; nasopharyngeal swab samples remained at Guillermo Almenara National Hospital to be analyzed independently by RT-qPCR. Handling of the nasopharyngeal swabs and saliva samples was conducted under recommended biosafety standards for respiratory viruses. The analyses were run blind, so that the personnel in each laboratory ignored of test results of the other laboratory. Finally, we evaluated the LOD of RCSMS by testing serial dilutions of synthetic RNA in a range of 5,000 to 1 viral copy per 10 μl RT-LAMP reaction, which included 2 μl of saliva inactivated with TCEP / EDTA and negative for SARS-CoV-2 in order to avoid degradation of the added RNA by saliva RNAses.

### Generation of synthetic viral RNA templates by PCR and in vitro transcription

To select the ideal detection targets for this study as well as to establish their optimal amplification and detection parameters, we generated a panel of synthetic RNA templates encoding the viral genes N (934 bp), E (532 bp), S (540 bp), from Nsp6 to Nsp8 (734 bp), from Nsp10 to Nsp12 (798 bp) and the human POP7 gene (406 bp, sample quality control). These RNA templates were generated via *in vitro* transcription from PCR products modified to contain the sequence of the T7 transcriptional promoter at their 5’ ends. The PCR reactions were performed using GoTaq G2 DNA polymerase (Promega) according to the conditions and cycling profile shown in Table 1. After resolving the amplification products on 1% agarose gels, the corresponding bands were cut and purified with help of the GeneJet Gel DNA Extraction Kit (ThermoScientific). The DNA concentration in the eluates was quantified using a Nanodrop 2000 spectrophotometer (ThermoScientific) and 100 ng DNA were used for the in vitro transcription reaction using the AmpliScribe T7-Flash Transcription Kit (Lucigen), following the manufacturer’s recommendations. The resulting RNA templates were purified with the GenElute kit (Sigma) and quantified with the Qubit 4.0 fluorometer using the RNA BR assay kit (Invitrogen). In addition, the integrity of the RNA was verified by denaturing agarose electrophoresis.

**Table 1:** Master-mix and program for SARS-CoV2 control template generation.

**Component**	**Initial concentration**	**Final concentration**	**Volume (μl)**
H_2_O	—	—	5.12 μl
5X Buffer	5X	1X	3 μl
dNTPs	10 mM	0.2 mM	0.3 μl
Primer 1	10 mM	0.33 mM	0.5 μl
Primer 2	10 mM	0.33 mM	0.5 μl
GoTaq G2 DNA Polymerase	5 U/μl	0.027 U/μl	0.08 μl
M-MLV Reverse Transcriptase	200 U/μl	6.67 U/μl	0.5 μl
Mastermix volume per reaction			10 μl
pCCI-4K-SARS-CoV-2-Wuhan-Hu-1			5 μl (5 ng)
Total volumen per reaction			15 μl
**Step**	**Temperature (°C)**	**Time (s)**	
Reverse Transcription	45	1200	
Initial Denat.	94	120	
35X Cycles	94	30	
	60	30	
	72	60	
Final Ext.	72	300	

### Inactivation of saliva samples and RNA extraction

Saliva samples containing 0.01 volumes of the 100X inactivation solution (0.25 M TCEP-HCl, 0.1 M EDTA and 1.15 N NaOH) were mixed and homogenized with a vortex for 10–15 sec, then heated for 10 min at 95°C and cooled on ice. For extraction controls from saliva and nasopharyngeal swab samples, RNA was extracted using the ReliaPrep Viral TNA Miniprep Kit, Custom (Promega) and the QIAamp Viral RNA Mini Kit (Qiagen).

### RT-LAMP amplification

The sets of six primers for the LAMP amplification of each viral gene (Table 2) were previously conjugated in a 10X master mix (Table 3). For the RT-LAMP reaction, a mixture of 0.2 μl Warmstart RTx Reverse Transcriptase and 0.5 μl Bst 3.0 DNA polymerase (NEB) were used, taking as input material 5 μl of purified RNA or 2 μl of inactivated saliva in a total volume of 10 μl (8 mM final concentration of MgSO4) and running the reactions for 30 min at 62°C.

**Table 2:** Sequences of primers, guide RNAs and probes used in the study.

Name	Sequence (5’ - 3’)	Application
RT-LAMP-N-F3	AACACAAGCTTTCGGCAG	RT-LAMP for SARS-CoV-2 N gene
RT-LAMP-N-B3	GAAATTTGGATCTTTGTCATCC	
RT-LAMP-N-FIP	TGCGGCCAATGTTTGTAATCAGCCAAGGAAATTTTGGGGAC	
RT-LAMP-N-BIP	CGCATTGGCATGGAAGTCACTTTGATGGCACCTGTGTAG	
RT-LAMP-N-LF	TTCCTTGTCTGATTAGTTC	
RT-LAMP-N-LB	ACCTTCGGGAACGTGGTT	
RT-LAMP-E-F3	CCGACGACGACTACTAGC	RT-LAMP for SARS-CoV-2 E gene
RT-LAMP-E-B3	AGAGTAAACGTAAAAAGAAGGTT	
RT-LAMP-E-FIP	ACCTGTCTCTTCCGAAACGAATTTGTAAGCACAAGCTGATG	
RT-LAMP-E-BIP	CTAGCCATCCTTACTGCGCTACTCACGTTAACAATATTGCA	
RT-LAMP-E-LF	TCGATTGTGTGCGTACTGC	
RT-LAMP-E-LB	TGAGTACATAAGTTCGTAC	
RT-LAMP-P-F3	TTGATGAGCTGGAGCCA	RT-LAMP human RNAse P (POP7) gene
RT-LAMP-P-B3	CACCCTCAATGCAGAGTC	
RT-LAMP-P-FIP	GTGTGACCCTGAAGACTCGGTTTTAGCCACTGACTCGGATC	
RT-LAMP-P-BIP	CCTCCGTGATATGGCTCTTCGTTTTTTTCTTACATGGCTCTGGTC	
RT-LAMP-P-LF	ATGTGGATGGCTGAGTTGTT	
RT-LAMP-P-LB	CATGCTGAGTACTGGACCTC	
E-gene-T7prom-F	AATTCTAATACGACTCACTATAGGGCTGGTGTTGAACATGTTACCTTCTTCATC	RT-PCR to generate template for in vitro transcription and generate synthetic RNA of the viral E gene (Promoter T7)
E-gene-T7prom-R	CCTATTACTAGGTTCCATTGTTC	
N-gene-T7prom-F	AATTCTAATACGACTCACTATAGGGCCAAATTGGCTACTACCGAAGAGCTAC	RT-PCR to generate template for in vitro transcription and generate synthetic RNA of the viral N gene (Promoter T7)
N-gene-T7prom-R	CACAGTTTGCTGTTTCTTCTGTCTCTGCGG	
POP7-gene-T7prom-F	AATTCTAATACGACTCACTATAGGGCTCTGAGATCTACATTCACGGCTT	RT-PCR to generate template for in vitro transcription and to generate synthetic RNA of the human POP7 gene (Promoter T7)
POP7-gene-T7prom-R	CAATAGTTACAGACCGCATACACAC	
C12a-FAM-Bio-Probe	/56-FAM/TTATTATT/3Bio/	CRISPR-Cas12a lateral flow readout
C12a-FAM-BHQ1-Probe	/56-FAM/TTATTATT/3BHQ_1/	CRISPR-Cas12a fluorescence readout
C12a-gRNA-N	UAAUUUCUACUAAGUGUAGAUCCCCCAGCGCUUCAGCGUUC	CRISPR-Cas12a gRNA for SARS-CoV-2 N gene
C12a-gRNA-E	UAAUUUCUACUAAGUGUAGAUGUGGUAUUCUUGCUAGUUAC	CRISPR-Cas12a gRNA for SARS-CoV-2 E gene
C12a-gRNA-P	UAAUUUCUACUAAGUGUAGAUAAUUACUUGGGUGUGACCCU	CRISPR-Cas12a gRNA for Human RNAse POP7 gene

**Table 3:** Proportions for master mix (10X) for RT-LAMP primers.

LAMP Primer (100uM en H2O)	Final concentration	Volume (μl)
F3	2 μM	20 μl
B3	2 μM	20 μl
FIP	16 μM	160 μl
BIP	16 μM	160 μl
LF	8 μM	80 μl
LB	8 μM	80 μl
Nuclease free water	-	480 μl
Total Volume		1000 μl

### CRISPR-Cas detection

**Fig 1:**
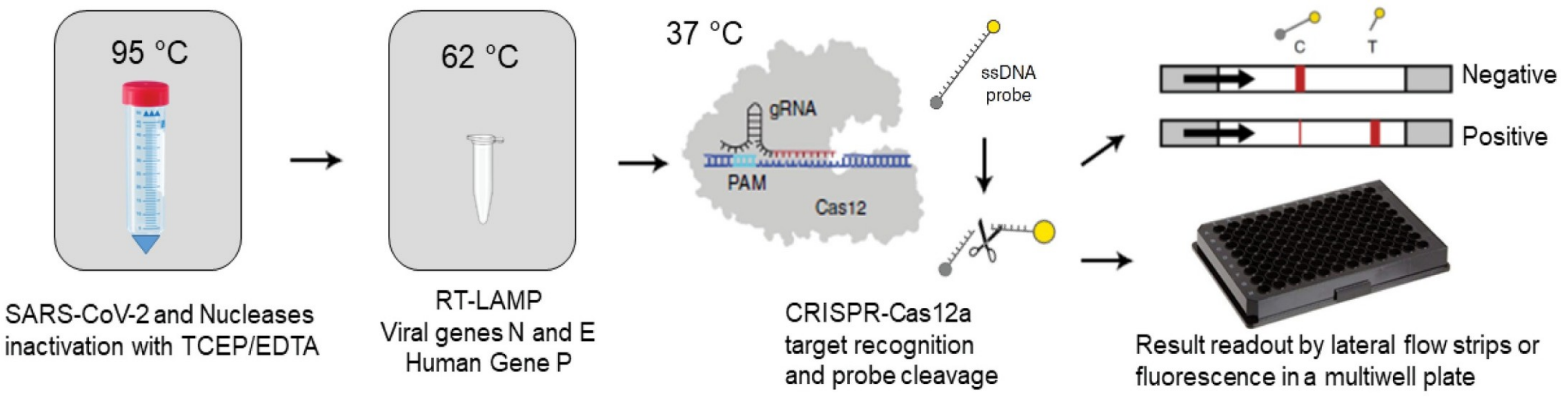
RCSMS detection workflow. Upon saliva treatment and inactivation with TCEP/EDTA, 2 μl of saliva sample are added to a 10 μl RT-LAMP reaction. A 2 μl aliquot of the RT-LAMP product is then mixed with the RNP complex consisting of Cas12 and RNA guides. Recognition of viral target sequences by the RNP complex triggers the collateral activity of Cas12a, resulting in the cleavage of ssDNA reporter probes. For immunochromatographic (qualitative) readout, a lateral flow strip is then inserted into the CRISPR-Cas12a reaction tube or well. Within two minutes, uncleaved reporter molecules flow and accumulate into the control capture line of the strip (C band in the image), whereas cleaved reporter molecules flow towards the target capture line of the strip (T band in the image), (adapted from Broughton et al. [9] and Patchsung et al. [18]. For fluorescence (quantitative) readout, CRISPR-Cas12a reactions are recorded in real-time over 10 min using an automated plater reader; cleaved reporter molecules yield a bright fluorescent signal.

For the formation of the ribonucleoprotein complex (RNP = Cas12a + guide RNA), the LbCas12a enzyme (NEB, 50 nM final concentration) was incubated with the guide RNA for each viral target (62.5 nM, final concentration) in 1X NEBuffer 2.1 for 30 min at 37°C. ssDNA probes (Table 2) were added to the mix at a concentration of 20 nM for fluorescence measurements (485 nm excitation, 528 nm emission) in a Cytation 7 plate reader (BioTek) 500 nM for lateral flow strips (Milenia GenLine Hybridetect, Milenia Biotech). For each fluorescence reaction, 2 μl of RT-LAMP product, 80 μl of 1X NEBuffer 2.1 and 20 μl of RNP were mixed per well of a 96-well plate (Costar). For lateral flow strips, 2 μl of RT-LAMP product and 20 μl of RNP were mixed and incubated in a 1.5 ml tube for 10 min at 37°C, and then 80 μl of pre-warmed Milenia GenLine Dipstick Assay Buffer buffer were added (for more details: https://www.milenia-biotec.com/en/tips-lateral-flow-readouts-crispr-cas-strategies/). For the sequences of all guide RNAs and CRISPR ssDNA probes, see Table 2. The positivity threshold was calculated as the ratio between signal and minimum fluorescence values (“fold change”) detected after 10 min of measurement. The reading and interpretation of the results in lateral flow strips is based on the appearance of diagnostic bands (Fig 1 and Table 4), while for fluorescence results, real-time or end-point measurements were made via automated reading on 96-well plates.

**Table 4:** Interpretation of results by immunochromatographic lateral flow strips.

Gene N o E	Gene P	Interpretation
+	+/-	Positive Result for SARS-CoV 2
-	+	Negative Result for SARS-CoV 2
-	-	Invalid Result

For reference on reading the lateral flow strips, see Fig 1

### Amplification and detection by RT-qPCR

The RT-qPCR of viral genes Orf1ab and N run at UPCH used the *in vitro* diagnostic (IVD) FTD-SARS-CoV-2 kit (Siemens) and QTower 3G real-time thermocyclers (Analytik-Jena). The RT-qPCRs performed at the Guillermo Almenara Irigoyen National Hospital used the IVD Viasure SARS-CoV-2 Real Time PCR Detection Kit (CerTest Biotec), which also amplifies the viral genes Orf1ab and N and the Rotor-Gene real-time PCR cycler (Qiagen). Diagnostic Ct threshold were set as recommended by each kit and according to WHO guidelines.

### Statistical analyses

A descriptive analysis of the sociodemographic and clinical characteristics of the population was carried out. These included measures of central tendency and dispersion that were calculated for the quantitative variables, as well as absolute and relative frequencies for the categorical variables. Likewise, the level of concordance (Cohen’s Kappa coefficient) between RT-qPCR and RCSMS was calculated for both analytical and clinical validations, as well as sensitivity, specificity, positive predictive value (PPV), negative predictive value (NPV), the area under the curve (AUC), accuracy of RCSMS, and PPV/ NPV simulations assuming various SARS-CoV-2 prevalence scenarios. A stratified analysis by time was also included (greater than 7 and less than 7 days of illness). The estimates included 95% confidence intervals (95% CI) with an exact binomial method, and a p value <0.05 was considered significant. Data visualization and analysis were done using custom R scripts and the ‘pROC’, ‘ggplot2’, ‘riskyr’, packages.

### Ethical considerations

Participants in the clinical validation stage were enrolled and sampled according to the ethics protocols approved by the INCOR EsSalud CEI (certificate 02/2021-CEI, S1 Appendix) and UPCH (SIDISI code 202099, S2 Appendix). All the participants read and signed informed consents (S3 Appendix) prior to providing samples for this study, and all the data from the patients (S1 Dataset) were fully anonymized.

## Results

### Amplification and detection of SARS-CoV-2 sequences by the RT-LAMP and CRISPR-Cas12a coupled reactions

Synthetic RNA fragments of the viral genes N (934 bp) and E (540 bp), and the human POP7 gene (406 bp), were selected and used as templates to optimize the conditions for RT-LAMP reactions (S1A and S1B Fig). The RT and LAMP reactions were run simultaneously in a single tube, taking ~10,000 copies of synthetic RNA fragments as starting material. Comparative evaluation of the reactions by real-time fluorescence and agarose gel electrophoresis revealed that optimal amplification of the three fragments occurred after 30 minutes at 62°C (S1C Fig).

**Fig 2:**
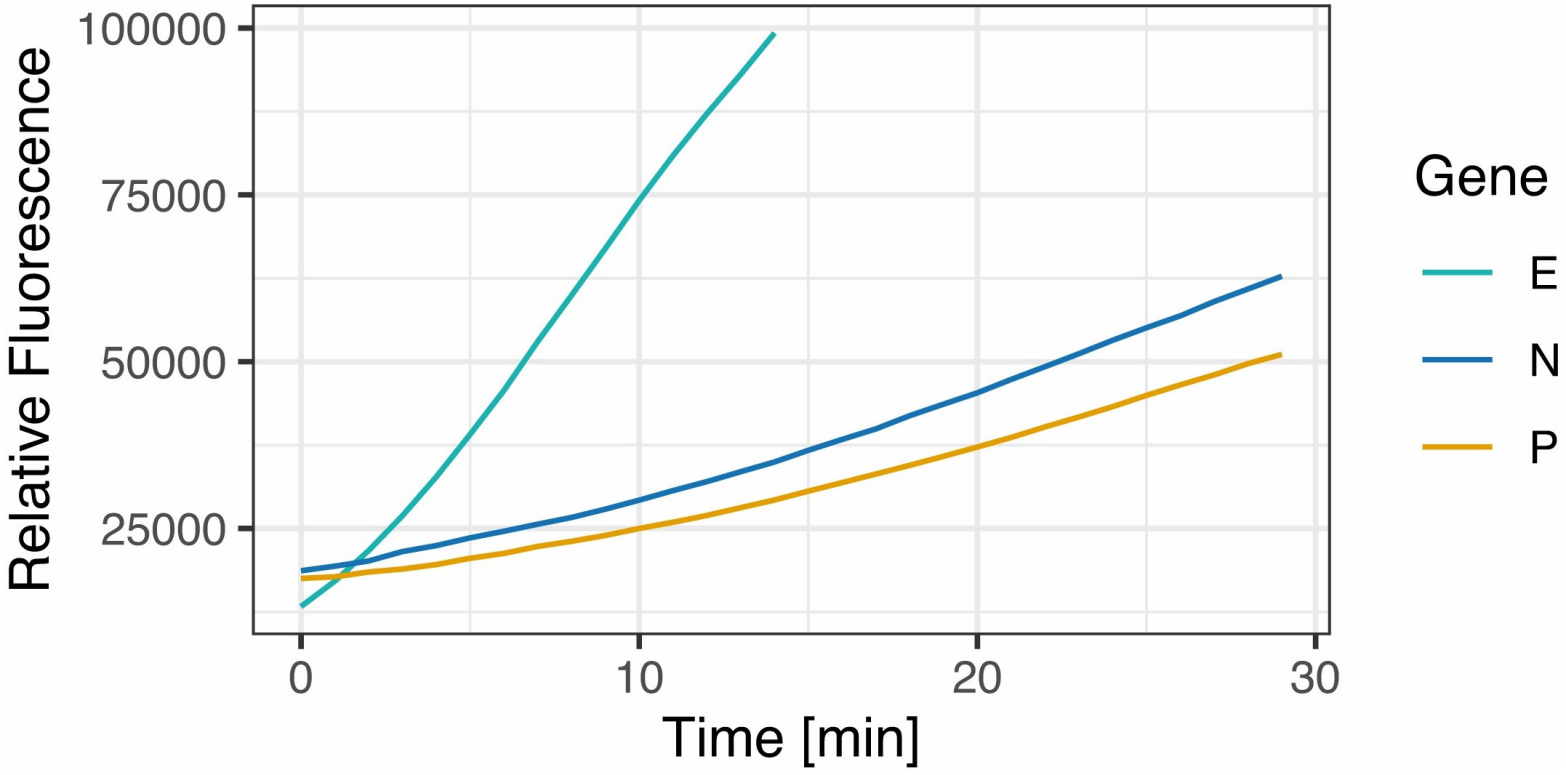
Fluorescent CRISPR-Cas12a-based detection of SARS-CoV-2 genes. Synthetic RNAs corresponding to the viral E, N and human POP7 genes were used as templates for RT-LAMP. The amplification products were then used as substrates for CRISPR-Cas12a reactions in the presence of fluorescent ssDNA reporter probes (485 nm excitation, 528 nm emission). Real-time fluorescence measurements of CRISPR-Cas12a reactions were performed separately for each gene, routinely every minute for 10 min at 37°C under the green channel of an automated plate reader. Signals obtained at every time point were normalized against the background signal detected at t = o; the curves represent the change in normalized fluorescence units (I-I_0_, y axis) over time (min, x axis). In the example shown, the red, green and yellow curves correspond to 30 min data collection for N, E and P genes, respectively. During such extended measurements, the E gene signal reached the instrument’s top detection limit after 15 min, whereas the N and P gene signals continued to grow at a lower rate.

**Fig 3:**
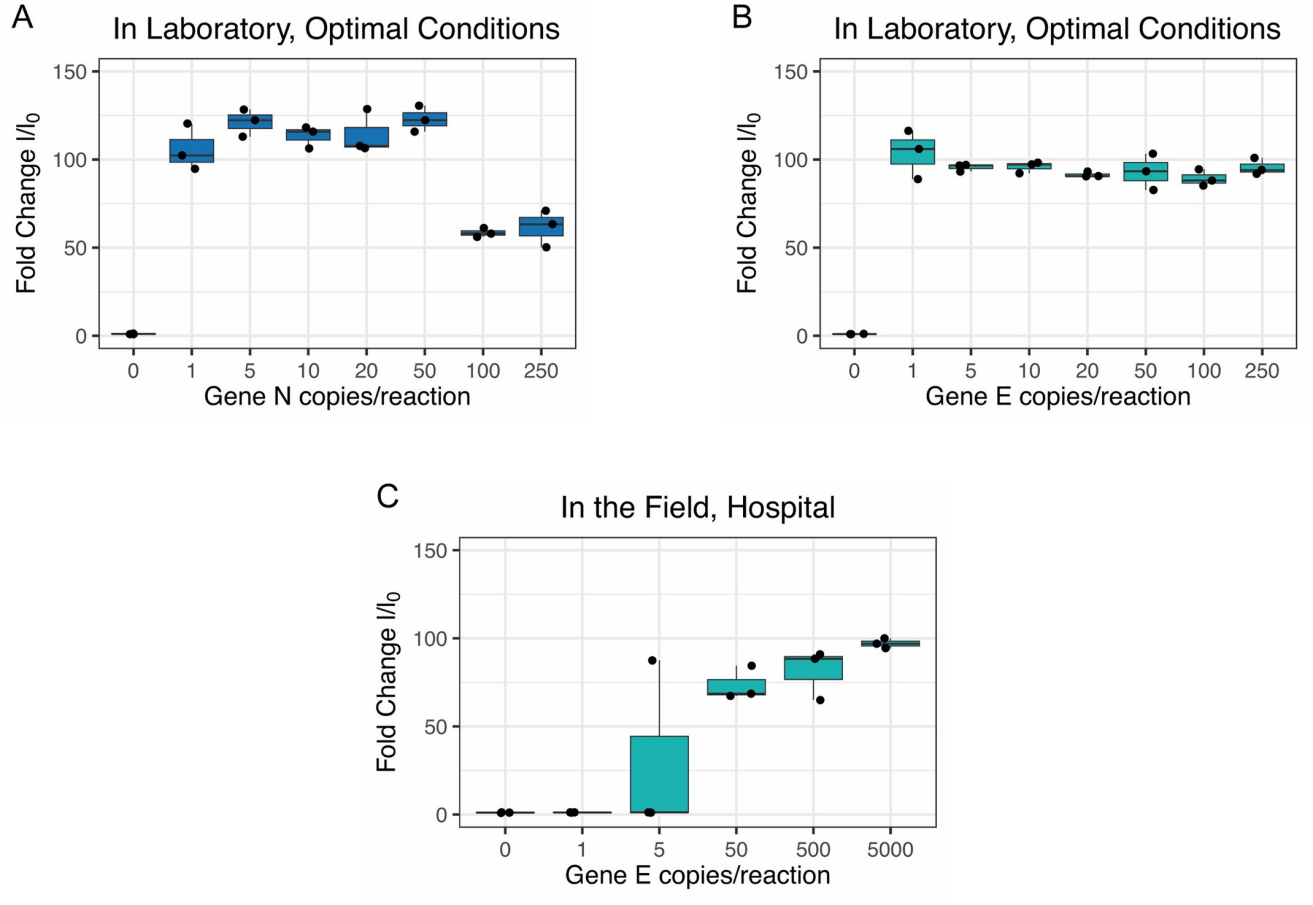
LOD estimations for analytical and clinical validations. Limits of detection were estimated from fluorescent CRISPR-Cas12a assays under laboratory and field conditions (lateral flow assays not shown here). The corresponding RT-LAMP reactions were performed in triplicate using serial dilutions of synthetic RNA templates in water or saliva. The graphs depict the fold change in fluorescence (y axis) vs the number of viral copies per RT-LAMP reaction (x axis). Fold change values (I/I_0_) were obtained by normalizing the fluorescent signal of the sample end-point (t = 10 min) against the background signal at t = 0. The lines between the dots represent the arithmetic mean with standard deviation for the three samples run for any given value of viral copies/reaction. (A) and (B) In an optimal laboratory setting, down to 1 viral copy per 10 μl RT-LAMP reaction was detected for both N and E genes. (C) Under field conditions, our test consistently detected down to 50 viral copies per 10 μl RT-LAMP reaction, with a single replicate detecting up to 5 viral copies per 10 μl reaction. See discussion for further details about LOD comparison with other studies.

The next step involved the detection of the amplified viral fragments using CRISPR-Cas12a. To this end, we used 2 μl of the RT-LAMP amplified products as substrate and 2 μl of RT-PCR products (generated from the same synthetic templates) as positive controls. The optimal incubation time for the formation of the RNP complex (i.e. Cas12a + guide RNA) was 10 min, and was stable up to 48 hours stored at 4°C. Cas12a-dependent detection of the E, N and POP7 genes worked optimally when the reaction run for 10 minutes at 37°C in the presence of 50 nM of RNP complex, formed by equimolar concentrations of Cas12a enzyme and RNA guides. Upon specific binding of the RNP complex to its amplified target viral genes, Cas12a-induced cleavage of the 8 bp reporter probe yielded optimal signals at 20 nM (fluorescent readout) and 500 nM (immunochromatography readout) (Fig 2). Fluorescence emission was stronger for gene N compared to the E and P genes. The minimum threshold of positivity of the fluorescence measurements was set at 20,000 Relative Fluorescence Units (RFU) after 10 min of reaction, and with a maximum of 100,000 RFU. On the other hand, the appearance of clear immunochromatographic signals on the lateral flow strips was consistently achieved after 2 min incubation of the strips in the reaction tubes. The limit of detection (LOD) for both viral genes was determined using serial dilutions of the synthetic RNA templates, covering the range from 250 to 1 viral copy per 10 μl RT-LAMP reaction. Remarkably, our assay was able of detect down to one copy of both N and E genes in fluorescence and immunochromatographic readings (Fig 3A and 3B and Table 5), showing excellent concordance and reproducibility between both detection formats.

**Table 5:** Limit of Detection (LOD) assay for analytical and clinical validation.

Copies / 10 μl reaction	Analytical Validation						Copies / 10 μl reaction	Clinical validation		
	Gene E			Gene N				Gene E		
250	+	+	+	+	+	+	5000	+	+	+
100	+	+	+	+	+	+	500	+	+	+
50	+	+	+	+	+	+	50	+	+	+
20	+	+	+	+	+	+	5	-	+	-
10	+	+	+	+	+	+	1	-	-	-
5	+	+	+	+	+	+	0	-	-	-
1	+	+	+	+	+	+				
0	-	-	-	-	-	-				

RT-LAMP reactions of 10 μl carried out in triplicate with synthetic RNA of the viral genes E and N, were used as substrate for CRISPR-Cas12a detection. Up to 1 viral copy per 10 μl reaction was detected in both genes during the analytical validation and consistently 50 viral copies per 10 μl reaction (including 2 μl of SARS-CoV-2 negative inactivated saliva) during clinical validation. A single replicate detected 5 viral copies per 10 μl reaction. See discussion for further details about LOD comparison with other studies.

**Fig 4:**
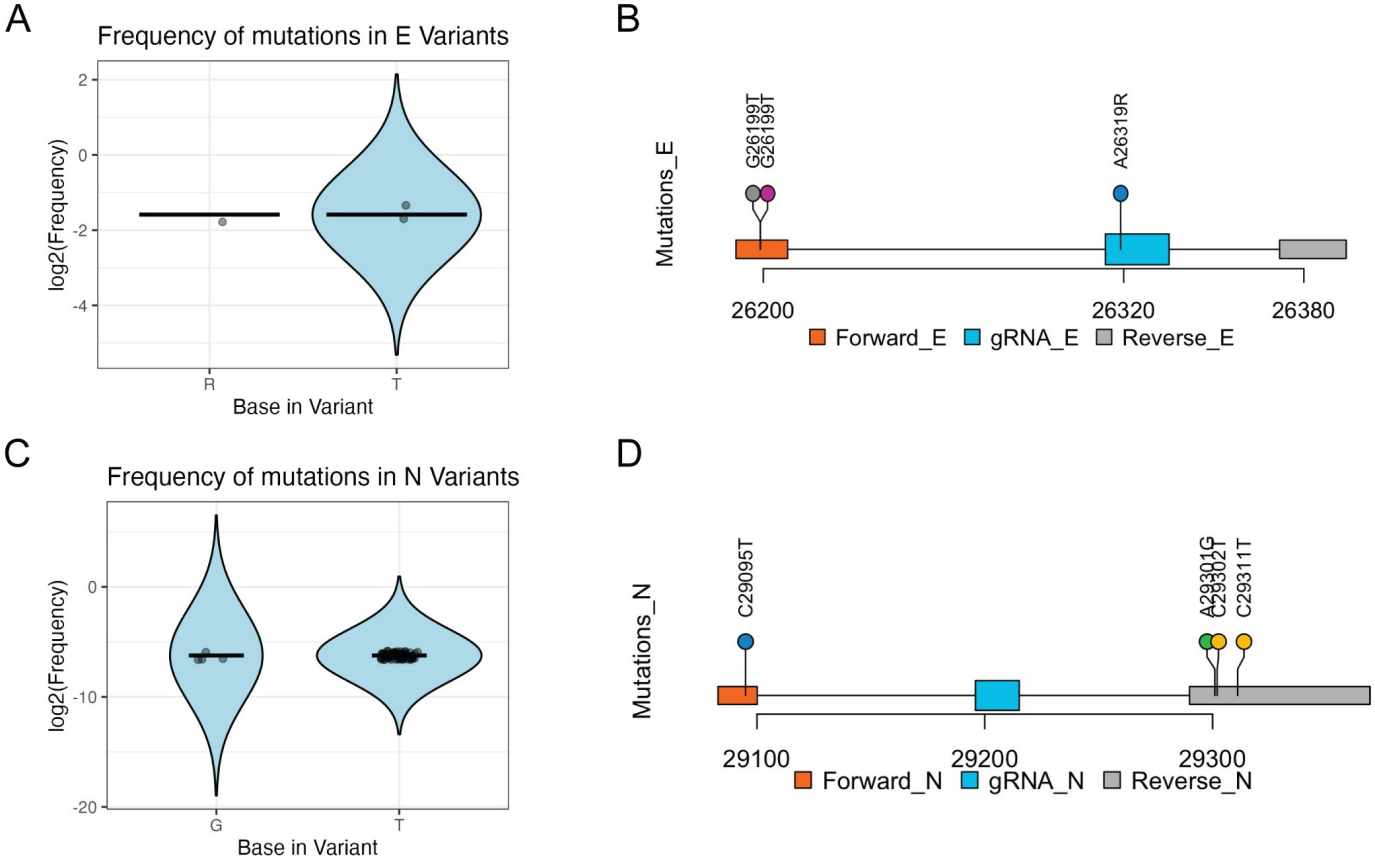
Analysis of nucleotide polymorphism within SARS-CoV-2 viral genomic RCSMS targets. Nucleotide polymorphisms—NCBI database, May 2023—in targeted regions for primers and gRNA sequences within SARS-CoV-2 N and E genes. (A) Log2 frequencies of the most common nucleotide polymorphism in gene N targeted regions. (B) Upper quantile polymorphisms in gene N targeted regions indicating position and nucleotide change. (C) Log2 frequencies of the most common nucleotide polymorphism in gene E targeted regions. (D) Upper quantile polymorphisms in gene E targeted regions indicating position and nucleotide change.

To rule out cross-detection of other related coronaviruses in the samples, we compared the sequences of RT-LAMP primers and guide RNAs used in this study with the corresponding regions in the viral E and N genes of different human coronaviruses (see Materials and methods). The analysis did not show significant similarities, except for the F3 LAMP primer of the E gene, which cannot produce cross-amplification by itself in a nested reaction. The appearance of SARS-CoV-2 variants worldwide makes it necessary to update all diagnostic and therapeutic methods based on the molecular recognition of viral genes and proteins. To this end, we analyzed 755 curated viral genomes from Peru available in NCBI database by May 2023 and examined all nucleotide polymorphisms within the E and N genes, which are the targets of this study (see Materials and methods). The analysis confirmed the high conservation of the viral genomes and the absence of mutational hot spots in the N and E genes corresponding to the binding sites of primers and guide RNA. However, we identified a few minor exceptions with very low frequencies (See Fig 4); for example, for the E gene, we identified two unique viral sequences containing each a G→T transition (singleton mutations) at the F3 primer-binding site, as well as one A→R mutation in the gRNA binding site. For the N gene, one mutation C→T in the F3 primer binding site, as well as two C→T and one A→G on the B3 primer binding site. None of these point mutations, however, disrupted the amplification efficiency of either E or N genes by RT-LAMP (see Discussion).

### Analytical validation of the RT-LAMP and CRISPR-Cas12a coupled method in Peruvian samples

**Fig 5:**
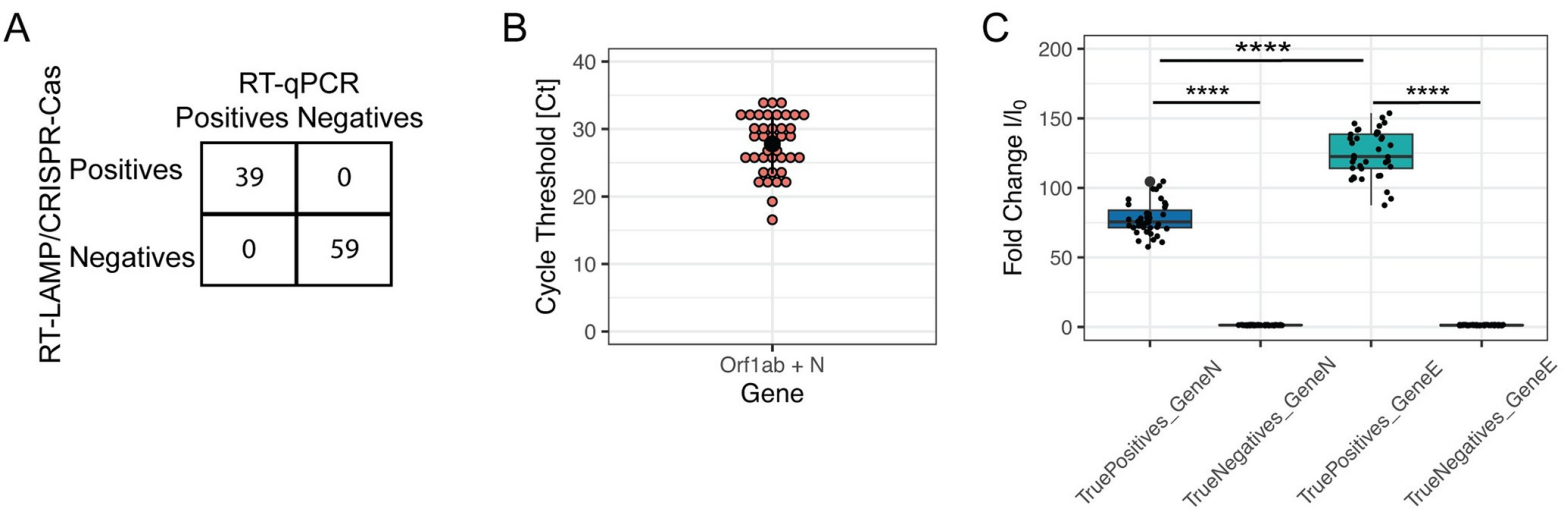
Retrospective analytical validation. The diagnostic performance of DETECTR was evaluated against RT-qPCR using 100 nasopharyngeal swabs from Peruvian patients, in triplicate and in blind. (A) 39 positive and 59 negative results were obtained with both methods, without false positives or false negatives. (B) Dot plot showing the cycle threshold values (Ct) of RT-qPCR for viral genes N and Orf1ab detected together in the same fluorescence channel. The horizontal line represents the arithmetic mean of all values with standard deviation. (C) Dot plot showing the fold change in fluorescence (y axis) for samples with the following DETECTR results: true positives for the E gene, true negatives for the E gene, true positives the N gene, and true negatives for the N gene (x axis). Fold change values (I/I_0_) were obtained by normalizing the fluorescent signal of the sample end-point (t = 10 min) against the background signal at t = 0. **** = p<0.0001 using the Kruskal-Wallis test, corrected with Dunn’s test, comparing all groups against the group of "true negatives" as a control group. Note that the fold change in fluorescence is significantly greater for the viral gene E compared to N.

For the retrospective analytical validation, we used 100 nasopharyngeal swabs, which were provided by the Instituto Nacional de Salud–INS, Perú. Saliva samples were not part of this analytical validation because the national repository of SARS-CoV-2 patient samples at the INS does not contemplate such samples. Viral RNA was extracted by conventional commercial methods and used as template for the amplification and detection of the viral genes E and N via the RT-LAMP and CRISPR-Cas12a coupled assay. Because of the absence of clinical and epidemiological data from the 100 INS samples, we introduced an additional verification step by RT-qPCR (Orf1ab and N genes) as an internal control to test the quality of the samples we received. Notably, all assays yielded 39 positive and 59 negative samples for SARS-CoV-2, plus two negative samples for the human RNase P gene (Fig 5). This result deviates from the expected 50 positive / 50 negative samples initially recorded by the INS at the moment they processed the samples by RT-qPCR. This inconsistency likely stems from the partial degradation of viral RNA in 11 positive samples and the degradation of total RNA in two negative samples, possibly caused by variable storage conditions and the transport of the samples at 4°C. Similar inconsistencies were independently reported by other research groups in Peru who received similar samples from INS to validate their own diagnostic kits. Moreover, three additional rounds of analyses reproduced these results, with only two positive samples yielding a negative result in one of their three replicates. Therefore, the corrected values for positivity and negativity for these 100 samples amounts to 39.8% and 60.2%, respectively. Importantly, the Kappa-Cohen index of 1.0 (95% CI: 1.0–1.0; p <0.00001) reaffirms the total agreement between the two detection methodologies (RT-LAMP/CRISPR-Cas12a coupled assay and RT-qPCR), with sensitivity values of 100% (95% CI: 91.0–100.0) and specificity of 100% (95% CI: 94.0–100.0), as well as a positive predictive value (PPV) of 100% (95% CI: 91.0–100.0) and a negative predictive value (NPV) of 100% (95% CI: 94.0–100.0) (Tables 6 and 8). Finally, as a measure of performance, the Area Under the Curve (AUC) of the Receiver Operating Characteristic (ROC) curve was calculated at 1.0 (95% CI: 1.0–1.0).

**Table 6:** Diagnostic performance measures of RT-LAMP / CRISPR-Cas in analytical and clinical validation.

Parameter	Analytical Validation		Clinical Validation					
			General		First Week		Second Week	
	Percentage (%)	IC 95%	Percentage (%)	IC 95%	Percentage (%)	IC 95%	Percentage (%)	IC 95%
Sensibility	100.0	0.91–1.0	96.5	93.7–99.2	93.4	90.8–98.8	98.7	97–99
Specificity	100.0	0.94–1.0	99	98.6–100.0	98.9	98.5–100.0	100	100–100
Positive Predictive Value	100.0	0.91–1.0	98.4	98.3–99.4	96.6	99.5–100	100	95.4–100
Negative Predictive Value	100.0	0.94–1.0	97.8	97.4–98.5	97.9	98.6–97	96.4	99.8–100
Area under the ROC curve	1.0	1.0–1.0	0.977	0.960–0.994	0.966	0.920–0.991	0.99	98.0–100

Measures of sensitivity, specificity, positive predictive value, negative predictive value, accuracy and area under the ROC curve of the RT-LAMP / CRISPR-Cas test, considering RT-qPCR as the “gold standard”.

### Clinical validation of RCSMS at two hospitals in Lima

Next, we set out to validate under real field conditions the performance of our RCSMS procedure, which combines the biosensing capabilities of RT-LAMP and CRISPR-Cas12a reactions with the addition of rapid and low-cost preparation of viral RNA from saliva samples via the TCEP/EDTA thermochemical method. Since one of the aims of this study aims to establish the use of saliva as initial sample, we first evaluated different conditions for the saliva inactivation assay. We tested different inactivation variables such as variations in the final concentration of TCEP/EDTA (0.5X, 1X, 2X and 5X), temperature (75°C, 80°C and 95°C) and time of inactivation (5, 10 and 15 minutes). From these initial optimization experiments, we found that efficient viral inactivation occurred when the saliva sample is treated at 95°C for 15 minutes. Moreover, we found that TCEP/EDTA concentrations between 0.5X and 2X yielded efficient viral inactivation and did not affect the subsequent RT-LAMP reaction (data not shown).

Next, we aimed to stablish the limit of detection (LoD) of our method. To this end we used serial dilutions (in saliva) of synthetic RNA, from 5,000 to 1 viral copies per 10 μl RT-LAMP reaction. We found the LoD for our method (either using fluorescence or immunochromatography) to be 50 viral copies per 10 μl RT-LAMP reaction (Fig 3C and Table 5), which is in close agreement with the LoD reported by DETECTR [8] and RT-qPCR [12–14].

Having established the LoD of our method, we started its clinical validation with real patient samples. To this end, we performed a prospective cross-sectional and observational study of RCSMS diagnostic precision using samples from 352 ambulatory patients over 18 years of age having symptomatology consistent with COVID-19, and who visited two of the main hospitals in Lima for SARS-CoV-2 detection. Their distributions according to gender, age group, time of illness and symptoms are shown in Table 7. Interestingly, we noticed a higher proportion of female participants (n = 200, 56.8%) and young adults (n = 246, 70.3%), with a mean age of 46.5 years and standard deviation of 14.4. The dominant duration of illness in the sample was one week of symptoms (n = 282, 80.1%) and the mean duration of symptoms was 4.7 days with a standard deviation of 3.5. Among the symptoms reported, sore throat predominated (n = 221, 62.8%), followed by headaches (n = 165, 46.9%). Twelve individuals (3.4% of the total) did not record their date of symptom onset; therefore, their results were excluded from the analysis stratified by time of illness.

**Table 7:** Clinical and epidemiological data of the samples used for clinical validation.

Sample characteristic		Quantity (n)	Percentage (%)
Sex	Male	152	43.1
	Female	200	56.8
Age group	Young	37	10.6
	Young Adult	246	70.3
	Elderly	67	19.1
Sick time	1 week	282	80.1
	2 weeks	58	16.5
	Not registered	12	3.4
Symptoms	Throat pain	221	62.8
	Fever	140	39.8
	Headache	165	46.9
	Anosmia	41	11.6
	Nasal congestion	84	23.9
	General discomfort	145	41.2
	Diarrhea	78	22.2
	Dysgeusia	37	10.5
	Chest pain	12	3.4
	Cough	52	14.7
RT-qPCR Result	Positive	138	39.2
	Negative	214	60.8
RT-LAMP / CRISPR-Cas Result	Positive	135	38.4
	Negative	217	61.6

352 individuals, their distributions according to gender, age group, time of illness and symptoms are shown.

**Fig 6:**
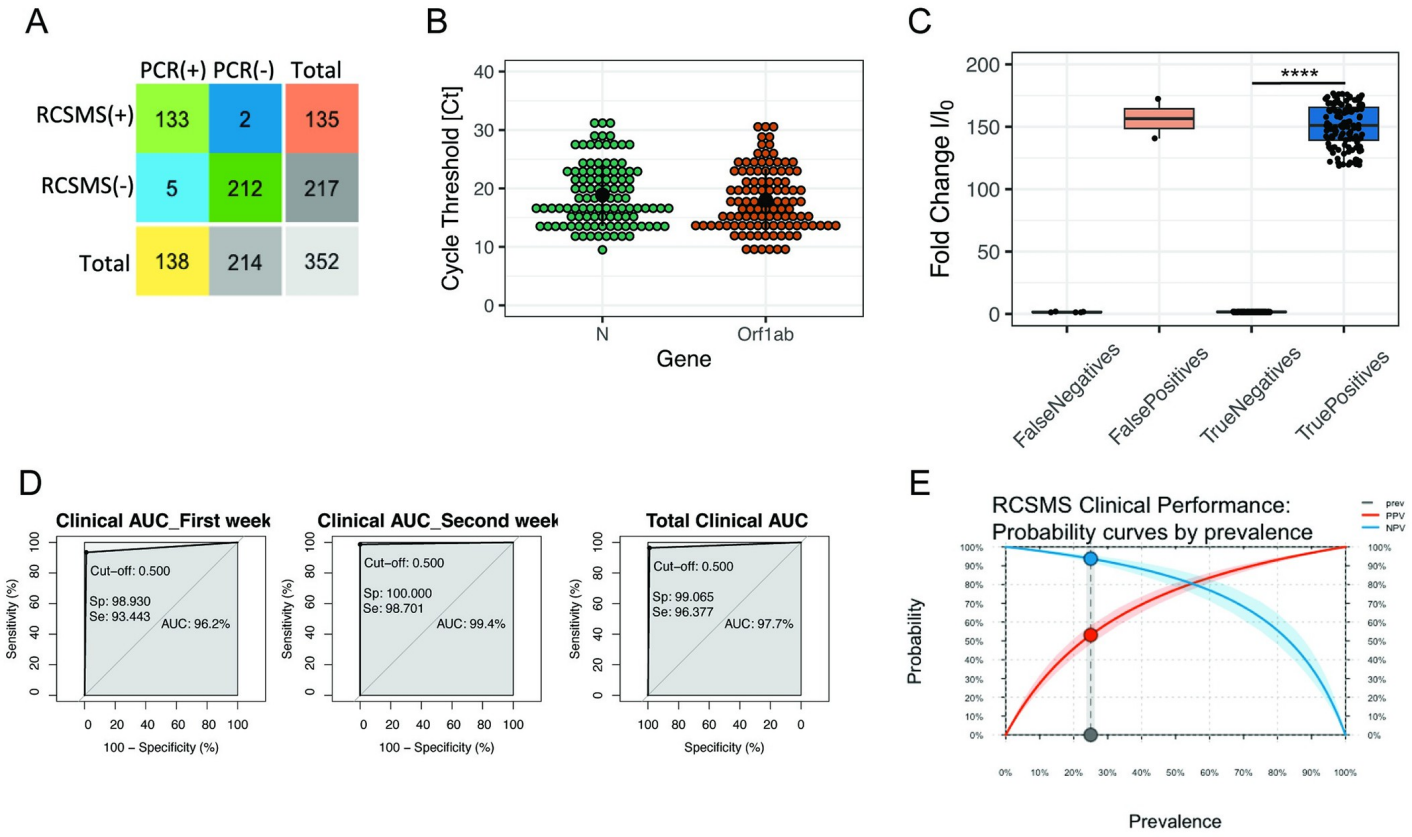
Clinical validation in Lima and post-test simulation of prevalence-dependent probability scenarios for PPV and NPV. The diagnostic performance of RCSMS was evaluated using 352 saliva samples from symptomatic patients at Guillermo Almenara and Edgardo Rebagliati Martins National Hospitals. A comparison was performed against RT-qPCR data obtained with nasopharyngeal swab RNAs from the same patients. (A) RT-qPCR produced 135 positive and 217 negative results, whereas RCSMS yielded 138 positive results, with false negatives and false positives (relative to RT-qPCR). (B) Dot plot showing the cycle threshold values (Ct) of RT-qPCR for viral genes N and Orf1ab detected in separate fluorescence channels. The horizontal line represents the arithmetic mean of all values with standard deviation. (C) Dot plot showing the fold change in fluorescence (y axis) for samples with the following RCSMS results for the E gene: true positives, true negatives, false positives, and false negatives (x axis). Fold change values (I/I_0_) were obtained by normalizing the fluorescent signal of the sample end-point (t = 10 min) against the background signal at t = 0. **** = p<0.0001 using the Mann-Whitney U test for comparison of true positives with true negatives. (D) Clinical AUC for the first and second week of testing and total; showing sensitivity (y axis) and specificity (x axis). (E) Expected variation of the positive and negative predictive values (PPV and NPV, respectively) for RCSMS according to different disease prevalence scenarios. X axis: prevalence of SARS-CoV-2 infection; y axis: PPV (blue line) and NPV (red line). In a scenario of >15% prevalence, both PPV and NPV >95%, whereas above 5% prevalence, the PPV >85%.

All saliva samples and nasopharyngeal swabs were analyzed in parallel via RCSMS and RT-qPCR. Of the 352 processed samples, 135 turned out positive by RCSMS (positivity = 38.4%) whereas 138 turned out positive by RT-qPCR (positivity = 39.2%) (Fig 6). Statistical analyses (Tables 6 and 8) yielded a prevalence of 29% (95% CI: 24.0–35.1), with a remarkable concordance between the two tests (Cohen’s Kappa coefficient of 0.96; 95% CI: 0.92–0.98; p <0.00001), 96.5% sensitivity (CI 95%: 93.7–99.2), 99.0% specificity (CI 95%: 98.6–100.0), PPV of 98.4% (CI 95%: 98.3–99.4), NPV of 97.8% (95% CI: 97.4–98.5) and the Area Under the Curve (AUC) of the receiver operating characteristic (ROC) curve of 0.977 (95% CI: 0.960–0.994). In total, seven incongruencies (1.99%) were recorded between the two diagnostic tests: two false positives (false positive rate, FPR = 1.5%) and five false negatives (false negative rate, FNR = 2.3%). To assess the performance of RCSMS under COVID-19 prevalence conditions different from those found in this study (~40%), we carried out a statistical simulation of PPV and NPV assuming known and fixed prevalence values in all scenarios (See Materials and methods). Our simulations predict a very good tolerance of RCSMS to changes in prevalence, with NPV values >98% in prevalence settings of up to 50%, and PPV values >80% in prevalence settings >10% (Fig 6).

**Table 8:** Results of analytical and clinical (field) evaluations using the RT-qPCR and DETECTR/RCSMS tests.

DETECTR/RCSMS		RT-qPCR		Total	Kappa (CI 95%; p value)
		Positive	Negative		
Analytical Validation	Positive	39	0	39	1.0 (1.0–1.0; p<0.00001) Perfect agreement
	Negative	0	59	59	
	Total	39	59	98	
Clinical Validation	Positive	133	2	135	0.95(0.92–0.98; p<0.00001) Almost perfect agreement
	Negative	5	212	217	
	Total	138	214	352	
Clinical validation (Week 1)	Positive	57	2	59	0.93 (0.87–0.97; p<0.00001) Almost perfect agreement
	Negative	4	185	189	
	Total	61	187	248	
Clinical validation (Week 2)	Positive	76	0	76	0.97 (0.9–1.0; p<0.00001) Almost perfect agreement
	Negative	1	27	28	
	Total	77	27	104	

The degree of concordance is shown using the Kappa-Cohen index considering a 95% confidence interval (95% CI).

Next, we asked whether the duration of the symptoms affected the diagnostic performance of RCSMS. We stratified the data by week 1 and 2 of symptoms. For the 242 patients in the first week of symptoms, a prevalence of 24.6% was obtained (95% CI: 19.5–30.5), with a Cohen’s Kappa coefficient between diagnostic tests of 0.93 (95% CI: 0.87–0.97; p <0.00001), 93.4% sensitivity (95% CI: 90.8–98.8), 98.9% specificity (95% CI: 98.5–100.0), PPV of 96.6% (95% CI: 99.5–100.0), NPV of 97.9% (95% CI: 98.6–97.0) and the Area Under the Curve (AUC) of the receiver operating characteristic (ROC) curve of 0.966 (95% CI: 0.920–0.991). For the 104 patients in the second week of symptoms, a prevalence of 74% (95% CI: 64.4–81.9) was observed, with a Cohen’s Kappa coefficient between diagnostic tests of 0.97 (95% CI: 0.9–1.0; p <0.00001), 98.7% sensitivity (95% CI: 97.0–99.0), 100% specificity (95% CI: 100.0–100.0), PPV of 100% (95% CI: 95.4–100.0), 96.4% NPV (95% CI: 99.8–100.0) and the Area Under the Curve (AUC) of the receiver operating characteristic (ROC) curve of 0.99 (95% CI: 0.98–1.0). In the second week, no false positives or false negatives were recorded, possibly due to the low representation of second-week patients in our study (most patients visit the hospital during the first week of symptoms). Notably, this stratified analysis suggests a potential increase of 5.3% in sensitivity and 1.1% in specificity of our RCSMS test between the first and second week of disease.

## Discussion

Peru consistently tops the list of countries most affected by COVID-19 worldwide. After three years and four pandemic waves, the country continues to lack adequate public health services, local biotechnology and the testing capacity to cover large urban and rural areas with limited access to current diagnostic technologies.

The present study describes the local implementation and clinical validation of RCSMS, a rapid and low-cost SARS-CoV-2 molecular test that combines the efficiency and robustness of the RT-LAMP and CRISPR-Cas12a reactions with the simplicity of the TCEP/EDTA thermochemical method for viral RNA preparation from saliva. We show that RCSMS detects SARS-CoV-2 RNA in ~40 min with a sensitivity of 96.33%, specificity of 99.65% and a LOD of 50 viral copies per 10 μl RT-LAMP reaction under real field conditions. The excellent diagnostic performance of RCSMS is further evidenced by the Cohen’s Kappa coefficients–relative to RT-qPCR- obtained under laboratory (1.00) or real field conditions (0.96) and is in good agreement with those obtained with other CRISPR-Cas-based tests, recently validated in hospitals worldwide [9, 18, 19].

Although nasopharyngeal swabs are a standard procedure for the collection of respiratory virus samples [3], their use raises the cost of testing, causes discomfort to the patient and increases the probability of transmission to and among sample collection personnel. Therefore, saliva tests are a significant step towards making COVID-19 diagnostics a routine task. Importantly, RCSMS implementation does not require biosafety level 2 laboratories because the lysis and inactivation of viral particles occur immediately after saliva collection, rendering samples non-infectious and keeping them safe from degradation by nucleases. Moreover, this viral and nuclease inactivation occurs at 95°C and the RT-LAMP reaction at 62°C. Therefore, the RNA extraction/inactivation and RT-LAMP steps cannot be performed in the same reaction.

On the other hand, LAMP is a low-cost DNA amplification method with simple visual readouts but prone to cross-contamination due to the high-efficiency of its nested-primer approach. Therefore, aerosol contamination during this step must be prevented by setting up reactions in a designated work area, separate from all other steps of the RCSMS protocol, and keeping the reaction tubes closed until used in the detection step. Notably, the CRISPR-Cas12a system adds precision to the system because it produces a signal only when the viral sequence is specifically recognized and cut by the Cas12a enzyme. This reaction sets off the indiscriminate cut -also by Cas12a- of hundreds of reporter molecules, thus amplifying the original LAMP signal with high resolution and intensity [20]. Moreover, there are other reported at home RT-LAMP based methods such as Prime CovidDetect [21] with a sensitivity and specificity of 100% but a LoD of 80 copies/μl; LUCIRA [22] with sensitivity and specificity of 91.1% and 93% respectively and no reported LoD; in addition other RT-LAMP detection from saliva [23] had 85% of specificity and a LoD of 100 copies per μl reaction. Along with, Alhamid, et al. [24] reported a 5 primer RT-LAMP assay with 99% and 96.7% of sensitivity and accuracy, respectively, and LoD of 20 copies/μl using viral RNA extracted from patient samples. More close to our test, a RT-LAMP amplification from inactivated saliva reached 39 copies per μl reaction [25]. Taken together, we believe that the enhanced target recognition provided by Cas enzymes consistently increases the likelihood of discriminating between true positives with low copy input and false positive amplification. On the other hand, the Limit of Detection (LoD) values primarily depend on the amplification step via RT-LAMP. In this context, the Cas step merely amplifies the retro-transcribed and amplified viral RNA signal through fluorescent or biotinylated probes.

The present design and clinical validation of RCSMS focused mainly on the detection of the E and N genes from SARS-Cov-2. Similar to what was reported for DETECTR [9, 19], we observed more efficient and consistent detection of the E gene, particularly evident from the fluorescence signal levels (Fig 5C) and kinetics (Fig 2). Interestingly, Brandsma et al. clinically validated DETECTR in three Dutch hospitals, but targeting only the N gene [19]. Moreover, the clinical validation of the Cas13a-based SHERLOCK kit found that the S gene (encoding the Spike protein) performed better than the Orf1ab and N genes [18]. We also attempted detection of the S gene (which encodes the spike protein) but obtained only spurious and highly variable results (data not shown). This discrepancy is likely explained by the fact that the Cas13a enzyme targets and cuts RNA instead of DNA as seen in Cas12a, and that Cas13a uses a guide RNA significantly longer (64 nucleotides) than the guide RNA used by Cas12a (41 nucleotides) [20, 26, 27], which together may affect recognition of the viral target sequence. Given that the Spike protein mediates cellular entry of the virus, it is also relevant to consider the role of positive selection on the fixation of S gene mutations associated with infectivity and immune-evasion, as seen in the VOC Gamma (P.1, first detected in Brazil/Japan), VOC Beta (B.1.351, first detected in South Africa), VOC Alpha (B.1.1.7, first detected in UK) variants [27–29]. For example, samples from a large number of UK COVID-19 patients were found to have S genes that could not be amplified by RT-qPCR due to the Δ69/Δ70 and N501Y mutations of the more transmissible and lethal Alpha variant [29, 30]. For diagnostic purposes, it is therefore advisable to target more conserved genetic sequences. Nonetheless, the versatility and specificity of RCSMS allows for rapid reprogramming simply by redesigning primers and guide RNAs to target new variant sequences.

The rapid evolution of SARS-CoV-2 genomes makes it necessary to continuously screen the population for virus variants to ensure that the performance of molecular tests is not affected by mutations. Our bioinformatic analysis of 755 curated viral genomes from Peruvian patients revealed the presence of a small number of mutations in one primer for the E gene and one primer for the N gene, on the primers F3, B3 and gRNAs evaluated. In addition, for our analysis we excluded 77 sequences for gene E and 38 sequences for the gene N which had sequencing issues in the areas analyzed: with this, 0.15% and 0% frequency of mutations in gRNAs E and N respectively. We acknowledge that mutations can occur, and this could be a weakness of our system; however, these point mutations occur at low frequency in the population as seen from our analysis above, do not significantly affect DNA amplification as reported by Tamanaha, et al. [31], and do not affect significantly the performance of our test, as evidenced by the 96.33% sensitivity and 99.65% specificity scores obtained here for RCSMS. Importantly, our test is flexible enough to redesign the primers and gRNAs in the event of higher frequency mutations appearing in new variants, and even be adapted for other respiratory viral testing from saliva (i.e. influenza).

The accuracy values of RCSMS (96.5% sensitivity and 99.0% specificity) are very similar to those reported with DETECTR (95% sensitivity and 100% specificity for the E gene; 95.5% sensitivity and 92.9% specificity for the N gene) [9, 19] and SHERLOCK (sensitivity 96% and specificity 100% for the S gene) [18]. Despite these high-performance values of our test, we detected very few false negatives (2 out of 352 samples = 0.6%) during the clinical validation of our test (Fig 6A) Because the Cq values of all 352 samples are very similar (S1 Dataset), we reason that a low viral copy number samples cannot explain these 2 false negative samples (Fig 6); instead, these samples probably were RCSMS negative because the RNA integrity was affected in these samples by sub-optimal saliva quality at the moment of its collection and storage.

Similar studies using Cas-based tests from nasopharyngeal swabs have reported as LoD 10 copies per μl reaction [9], 50 and 42 copies per 25 μl reaction [18, 19], while our RCSMS consistently detected 50 copies per 10 μl reaction in triplicate assays, occasionally reaching detection of 5 copies per 10 μl reaction in one replicate. We hypothesize that the difference between our LoD and the reported by DETECTR [9] may be caused mainly due to the different size of the RNAs employed in our study and Broughton’s [9]. Specifically, we used synthetic RNA fragments of 536 and 930 bases, while the Broughton study used total RNA extracted from patient samples, and whose lengths were approximately 30 Kilobases long. The longer RNA fragment likely led to the formation of RNA secondary structures, which can affect binding and recognition to the BST polymerase in the RT-LAMP reaction, affecting as a result the amplification of the target SARS-CoV-2 sequence. In addition, given that the RNA quantification by RT-qPCR is more sensitive than fluorescence, this difference of LOD maybe due to an underestimation of the concentration of the synthetic RNA fragments used in our studies, which were quantified with a fluorometer and not with RT-qPCR. Consistent with this idea, it has been reported that RNA and DNA concentrations quantified by Qubit fluorescence (which we use here) is often underestimated compared to other quantification methods (such as UV or qPCR) [32]. This underestimation could range from 10 to 50% at the conditions used in our study [32]. Therefore, this underestimated RNA concentration likely explains why our analytical LOD is lower than those reported by other studies where the RNA products were quantified by UV or qPCR. This should be considered when comparing LODs reported by other studies that use different RNA quantification methods.

While we had no access to clinical samples containing other human coronaviruses, the ability of DETECTR to detect the E and N genes of SARS-CoV-2 (and not those of other related human coronaviruses) has been demonstrated *in silico* and *in vitro* by Broughton et al. [9].

We found that the performance of RCSMS was not affected by minor variations in experimental conditions. Specifically, TCEP/EDTA solution doesn’t compromise RT-LAMP reaction when used at concentrations between 0.5X to 2X, 10 μl LAMP reactions performed efficiently between 60 and 65°C and CRISPR-Cas detection was not compromised when using half the concentration of Cas12a or guide RNAs, reducing the reaction time to 5 min, or varying the input DNA volume between 0.2 and 3 μl. Similarly, a 6-fold increase in the concentration of fluorescent probes or a 0.1X variation in the concentration of biotinylated probes had no appreciable effect on detection (data not shown). We also tested different saliva inactivation variables and found that efficient viral inactivation was achieved at 95°C after 15 minutes.

Recently, novel CRISPR-Cas detection systems have been developed (such as STOPCovid [33] and Optima-dx [34]), which use thermostable version of Cas enzymes that allow to perform single-step reactions for SARS-CoV-2 viral RNA detection. While these thermostable Cas enzymes can simplify the detection assay into a single step, unfortunately they are not commercially available, which limits their application for massive use in developing countries. Despite that our RCSMS test is a two-step assay, its components (specifically the Cas12 enzyme) are widely commercially available. This is a significant advantage of our test over the more recent one-step assays because its production and massification can be done more easily, especially in developing countries, which is the motivation of the development of this test.

Sensitivity and specificity are inherent properties of any test and therefore should not change significantly between populations. However, the usefulness of a test, inferred from its predictive value, varies according to the true prevalence of the disease in a population. This is particularly relevant for COVID-19, where prevalence follows the evolution of complex geographical and temporal patterns of transmission, owing to demographic structure, human travel and migration. We estimated the predictive value of RCSMS under different prevalence settings to determine adequate scenarios for its use. Generally, the NPV of diagnostic tests is expected to drop in high prevalence settings, as the number of false negatives increase [35]. Nonetheless, our statistical simulation for RCSMS using the data (Fig 6F and 6G), predicts excellent NPVs >98% for prevalence scenarios as low as 1% and as high as 50%. Ensuring a low rate of false negatives (positive individuals being deemed negative) is especially desirable considering that COVID-19 is a serious, largely asymptomatic, and contagious disease, for which early measures are advisable. Thus, a negative RCSMS result would maintain a very good level of accuracy in high prevalence settings (i.e. at a hospital with a high proportion of COVID-19 patients), even as the prevalence sinks due to the natural progression of the epidemic curve. Moreover, these data indicate that RCSMS could be used with confidence to rule out infection in low prevalence settings such as airports and land travel facilities, schools, universities, sporting or cultural events, as well as religious services and working environments with high flow of personnel, where ensuring negativity is important.

Our simulation also obtains adequate PPVs >91% and >82% for prevalence scenarios >20% and 10%, respectively. These PPV estimates are consistent with the results from our field study, where prevalence reached almost 30%, strengthening the conclusion that RCSMS is particularly well suited for the identification of positive cases during acute pandemic phases, such as the current second waves hitting Perú, India and Brazil. A sharp decrease of PPV in low prevalence settings is common to all diagnostic tests because of the higher probability of obtaining false positives [36]. Notably, according to our simulation, RCSMS is expected to perform reasonably well (PPV>70%) even at a 5% prevalence scenario. As the pandemic recedes and the COVID-19 prevalence in the population decreases below 5%, it will be advisable to complement positive results using other molecular tests as well as diagnostic criteria (symptoms, oxygen saturation, chest X-ray/CT scan). Altogether, our data strongly suggest that RCSMS is sensitive enough to avoid missing infected individuals in high prevalence settings, and specific enough to maintain a very low proportion of erroneously diagnosed cases in low prevalence settings.

In conclusion, the present study integrates two methodologies that had been separately applied to COVID-19 testing: 1) the use of inactivated saliva as starting material for viral gene amplification [35] and 2) the use of DETECTR as a SARS-CoV-2 biosensor [9]. The resulting molecular test, RCSMS, can be easily implemented at the primary care level and diagnostic laboratories alike, using a simple lateral flow readout. To our knowledge, this is the first *clinical validation* of a CRISPR-Cas-based test for COVID-19 in Latin America using saliva, and one of the few of its type developed worldwide [36–39]. Noteworthy, an Argentinian group recently reported a DETECTR system [40] that uses nasopharyngeal swabs and fluorescence readouts. Within this group of newly validated CRISPR-Cas-based tests for the detection of SARS-CoV-2, RCSMS has the potential of becoming an important tool for the massification of COVID-19 molecular tests in Peru and neighboring countries.

## Supporting information

S1 Fig PCR and RT-LAMP templates and products. Visualization of various RT-PCR products, *in vitro* synthetized RNA templates and RT-LAMP products used for RCSMS standardization, on 1% agarose gels stained with ethidium bromide. (A) RT-PCR products used as DNA templates for the generation of *in vitro* transcribed RNAs; Lane 1: 931 bp viral N gene fragment, Lane 2: 557 bp viral E gene fragment, Lane 3: 653 bp viral S gene fragment; Lane 4: 751 bp viral Nsp6 to Nsp8 gene fragment, Lane 5: 815 bp viral Nsp10 to Nsp12 gene fragment, Lane 6: 428 bp human POP7 RNAse gene fragment, Lane 7: RT-PCR Negative control, Lane L: GenRuler 100bp Plus DNA Ladder (Thermo). (B) *in vitro* generated RNA templates; Lane 1: viral N gene; Lane 2: viral E gene; Lane 3: viral S gene; Lane 4: viral Nsp6 to Nsp8 gene; Lane 5: viral Nsp10 to Nsp12 gene; Lane 6: human RNAse POP7 gene. (C) Titration of E gene RNA input in 10 μl RT-LAMP reactions to obtain the characteristic LAMP ladder product; Lane L GenRuler 100bp Plus DNA Ladder (Thermo); Lane 1: reaction with 0 copies; Lane 2: reaction with 1 copy; Lane 3: reaction with 5 copies, Lane 4: reaction with 10 copies; Lane 5: reaction with 20 copies; Lane 6: reaction with 50; Lane 7: reaction with 100 copies; Lane 8: reaction with 250 copies.
(TIF)

S1 Appendix INCOR ethics committee approval. Participants in the clinical validation stage were enrolled and sampled according to the ethics protocols approved by the INCOR EsSalud of Peru. CEI certificate 02/2021-CEI.
(PDF)

S2 Appendix UPCH ethics committee approval. Participants in the clinical validation stage were enrolled and sampled according to the ethics protocols approved by Universidad Peruana Cayetano Heredia (UPCH) ethics committee. SIDISI code 202099.
(PDF)

S3 Appendix Participant’s informed consent. All the participants read and signed informed consents prior to providing samples for this study.
(PDF)

S1 Dataset Data for RCSMS validation. All the collected clinical data from the patients (S1 Dataset) were fully anonymized.
(XLSX)
