# Supplementary figures and images for: Clinical validation of RCSMS: A rapid and sensitive CRISPR-Cas12a test for the molecular detection of SARS-CoV-2 from saliva
Source: PLoS One. 2024 Mar 25;19(3):e0290466. doi: 10.1371/journal.pone.0290466 (PMC10962837; doi:10.1371/journal.pone.0290466)

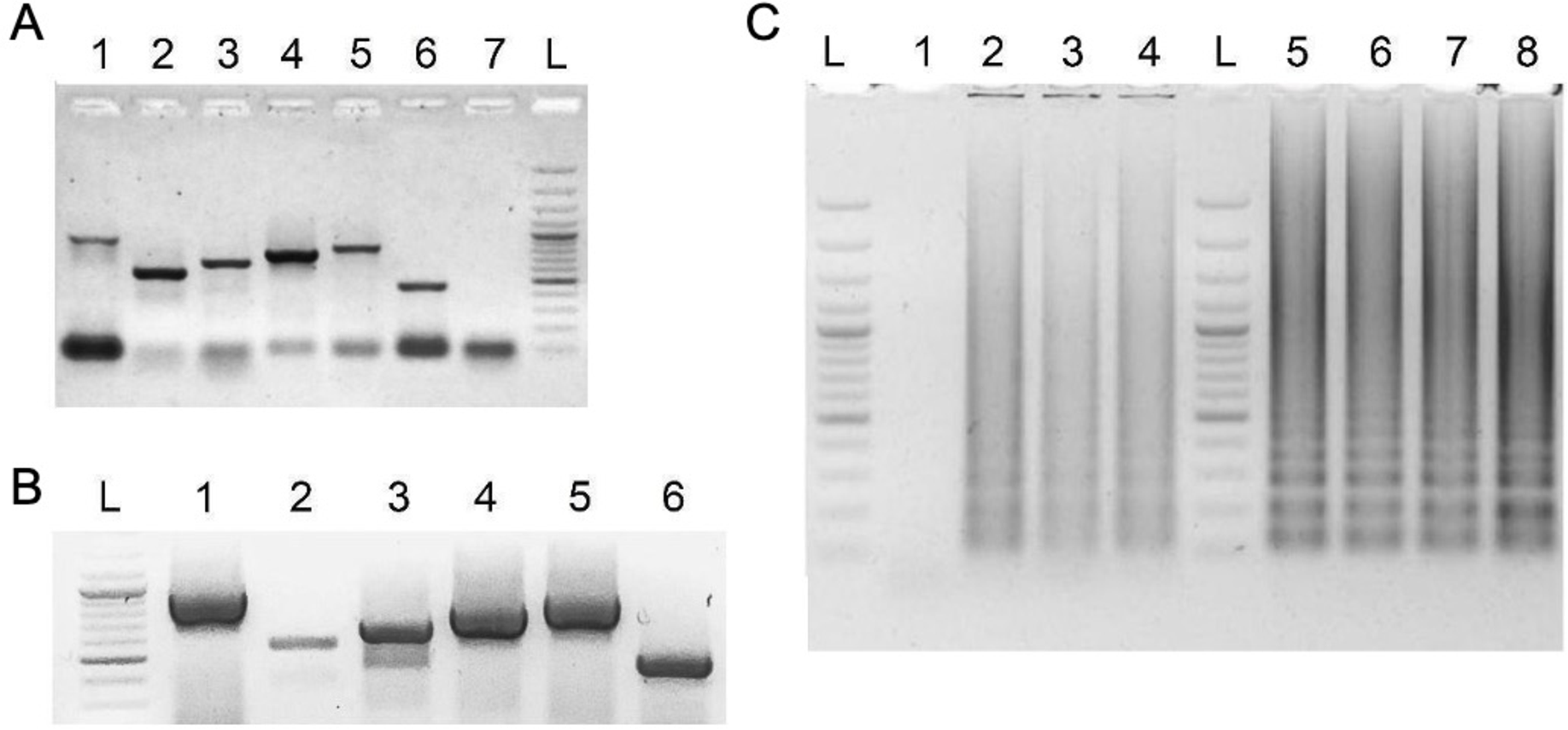

Supplement: S1 Fig — Visualization of various RT-PCR products, in vitro synthetized RNA templates and RT-LAMP products used for RCSMS standardization, on 1% agarose gels stained with ethidium bromide. (A) RT-PCR products used as DNA templates for the generation of in vitro transcribed RNAs; Lane 1: 931 bp viral N gene fragment, Lane 2: 557 bp viral E gene fragment, Lane 3: 653 bp viral S gene fragment; Lane 4: 751 bp viral Nsp6 to Nsp8 gene fragment, Lane 5: 815 bp viral Nsp10 to Nsp12 gene fragment, Lane 6: 428 bp human POP7 RNAse gene fragment, Lane 7: RT-PCR Negative control, Lane L: GenRuler 100bp Plus DNA Ladder (Thermo). (B) in vitro generated RNA templates; Lane 1: viral N gene; Lane 2: viral E gene; Lane 3: viral S gene; Lane 4: viral Nsp6 to Nsp8 gene; Lane 5: viral Nsp10 to Nsp12 gene; Lane 6: human RNAse POP7 gene. (C) Titration of E gene RNA input in 10 μl RT-LAMP reactions to obtain the characteristic LAMP ladder product; Lane L GenRuler 100bp Plus DNA Ladder (Thermo); Lane 1: reaction with 0 copies; Lane 2: reaction with 1 copy; Lane 3: reaction with 5 copies, Lane 4: reaction with 10 copies; Lane 5: reaction with 20 copies; Lane 6: reaction with 50; Lane 7: reaction with 100 copies; Lane 8: reaction with 250 copies. (TIF) [file pone.0290466.s001.tif]
