# Supplementary material for: Clinical validation of RCSMS: A rapid and sensitive CRISPR-Cas12a test for the molecular detection of SARS-CoV-2 from saliva
Source: PLoS One. 2024 Mar 25;19(3):e0290466. doi: 10.1371/journal.pone.0290466 (PMC10962837; doi:10.1371/journal.pone.0290466)
Supplement: S1 Appendix — Participants in the clinical validation stage were enrolled and sampled according to the ethics protocols approved by the INCOR EsSalud of Peru. CEI certificate 02/2021-CEI. (PDF) [file pone.0290466.s002.pdf]

certificate of approval

02/2021-CEI

January 18th of 2021

Research Ethics Cometee

Instituto Nacional Cardiovascular “Carlos Alberto Peschiera Carrillo” – INCOR

Jr. Coronel Felix Cipriano Zegarra 417 – Jesus Maria – Lima – Peru

Phone: 4111560 Email: comitedeeticaincor@gmail.com

CERTIFIED

That the Research Ethics Cometee from Instituto Nacional Cardiovascular “Carlos Alberto Peschiera Carrillo”, in the meeting of 07/01/2021, certificate 01-2021, has evaluated the proposal referred to the research, and reviewed the rectification of the Protocol observations:

Title: **“validación de campo de una prueba diagnostica de SARS-CoV-2 mediante CRISPR-Cas en muestras de saliva en sujetos sintomáticos de Lima, 2021”**

Approved documents:

- Research protocol

That in this study:

- the necessary requirements of suitability of the project in relation to the objectives of the study are met.
- the capacity of the researcher and the available means are adequate to carry out the study.
- The ethical precepts formulated in the Declaration of Helsinki of the World Medical Association on Ethical Principles for Research in Human Beings and in its subsequent revisions are complied with, as well as those required by the applicable legal standard based on the characteristics of the study.

That is why the committee **reports favourably** on the realization of said project by the researcher Joaquin Abugattas-Nunez Del Prado, as principal investigator.

This approval is valid for 12 months, which expires on 01/19/2022, and the renewal of the approval must be requested, if applicable, thirty days in advance.

Lima, 18th of January of 2021

SIGNATURE

Dra. Cecilia Cuevas De La Cruz

President of Research Ethics Cometee INCOR

EsSalud

Instituto Nacional Cardiovascular

Certificado de Aprobación
02/2021-CEI
18 de enero de 2021

Comité de Ética en Investigación
Instituto Nacional Cardiovascular "Carlos Alberto Peschiera Carrillo" - INCOR
Jr. Coronel Félix Cipriano Zegarra Nº 417 - Jesús María - Lima - Perú.
Teléfono: 4111560 Correo electrónico: comitedeeticaincor@gmail.com

CERTIFICA

Que el Comité de Ética en Investigación del Instituto Nacional Cardiovascular "Carlos Alberto Peschiera Carrillo", en su reunión del día 07/01/2021, acta 01-2021, ha evaluado la propuesta del investigador referida al estudio, y posteriormente ha revisado la subsanación de las observaciones del Protocolo:
Título: **"Validación de campo de una prueba diagnóstica de SARS-CoV-2 mediante CRISPR-CAS en muestras de saliva en sujetos sintomáticos de Lima, 2021"**

Documentos aprobados:
- Protocolo de investigación

Que en este estudio:
- Se cumplen los requisitos necesarios de idoneidad del proyecto en relación con los objetivos del estudio.
- La capacidad del investigador y los medios disponibles son adecuados para llevar a cabo el estudio.
- Se cumplen los preceptos éticos formulados en la Declaración de Helsinki de la Asociación Médica Mundial sobre principios éticos para las investigaciones en seres humanos y en sus posteriores revisiones, así como aquellos exigidos por la normativa legal aplicable en función de las características del estudio.

Es por ello que el Comité **informa favorablemente** sobre la realización de dicho proyecto por el investigador ***Joaquín Abugattas Núñez del Prado***, como investigador principal.

Esta aprobación tiene una vigencia de 12 meses, que vence el 19/01/2022, debiendo solicitar la Renovación de aprobación, de ser el caso, con treinta días de anticipación.

Lima, 18 de enero de 2021

Dra. Cecilia Cuevas De La Cruz
Presidenta del Comité de Ética en Investigación INCOR
