# Supplementary material for: Clinical validation of RCSMS: A rapid and sensitive CRISPR-Cas12a test for the molecular detection of SARS-CoV-2 from saliva
Source: PLoS One. 2024 Mar 25;19(3):e0290466. doi: 10.1371/journal.pone.0290466 (PMC10962837; doi:10.1371/journal.pone.0290466)
Supplement: S2 Appendix — Participants in the clinical validation stage were enrolled and sampled according to the ethics protocols approved by Universidad Peruana Cayetano Heredia (UPCH) ethics committee. SIDISI code 202099. (PDF) [file pone.0290466.s003.pdf]

Machine Translated by Google

CONSTANCY

The President of the Institutional Committee of Research Ethics (CIEI) of the Peruvian University Cayetano Heredia certifies that the institutional research ethics committee expeditiously approved the **AMENDMENT/MODIFICATION** of the research project indicated to continuation.

Title of the project : **"Implementation of new rapid and low-cost tests for the massive molecular diagnosis of SARS-CoV-2 without RT-PCR".**

Inscription code : **202099**

Principal investigator : **Malaga Trillo, Edward.**

The **amendment/modification** corresponds to the following documents:

1. **Investigation protocol,** version 2.0 received on January 20, 2021.
2. **Informed consent,** version received on January 20, 2021.

Lima, January 20, 2021.

COMITE INSTITUCIONAL DE ETICA-CIE
UNIVERSIDAD PERUANA CAYETANO HEREDIA
DUICT

Dr. Frine Samalvides Cuba
President
Institutional Research Ethics Committee

/ cht

Av. Honorio Delgado 430, SMP 15102
Apartado postal 4314
(511) 319-0000 anexo 201352
duict@oficinas-upch.pe
www.cayetano.edu.pe

CONSTANCIA

El Presidente del Comité Institucional de Ética en Investigación (CIEI) de la Universidad Peruana Cayetano Heredia hace constar que el comité institucional de ética en investigación aprobó de manera expedita la **ENMIENDA/MODIFICACIÓN** del proyecto de investigación señalado a continuación.

Título del Proyecto : **"Implementación de nuevos tests rápidos y de bajo costo para el diagnóstico molecular masivo de SARS-CoV-2 sin RT-PCR".**

Código de inscripción : **202099**

Investigador principal : **Málaga Trillo, Edward.**

La **enmienda/modificación** corresponde a los siguientes documentos:

1. **Protocolo de investigación,** versión 2.0 recibida en fecha 20 de enero del 2021.
2. **Consentimiento informado,** versión recibida en fecha 20 de enero del 2021.

Lima, 20 de enero del 2021.

COMITÉ INSTITUCIONAL DE ÉTICA-CIE UNIVERSIDAD PERUANA CAYETANO HEREDIA DUICT

Dra. Frine Samalvides Cuba
Presidenta
Comité Institucional de Ética en Investigación

/*cbt*
