# Supplementary material for: Clinical validation of RCSMS: A rapid and sensitive CRISPR-Cas12a test for the molecular detection of SARS-CoV-2 from saliva
Source: PLoS One. 2024 Mar 25;19(3):e0290466. doi: 10.1371/journal.pone.0290466 (PMC10962837; doi:10.1371/journal.pone.0290466)
Supplement: S3 Appendix — All the participants read and signed informed consents prior to providing samples for this study. (PDF) [file pone.0290466.s004.pdf]

Machine Translated by Google

Annex 2: Informed Consent

Study: "Field validation of a diagnostic test for SARS-CoV-2 using CRISPR CAS in saliva samples in symptomatic subjects from Lima, 2021"

Research Institutions:
Peruvian University Cayetano Heredia
Social Health Insurance

Principal investigator:
Joaquin Abugattas Nunez del Prado

Introduction: Your participation in this study is completely voluntary. You can decide not to participate and this decision will not affect your treatment by your doctor.

1. Why is this study being done?

The Universidad Peruano Cayetano Heredia (UPCH) and the Social Health Security (EsSalud) are conducting research to validate a new test developed in Peru to detect the SARS-CoV-2 virus that causes the COVID-19 disease by evaluating a saliva sample.

2. How will your participation be?

After receiving the information about the study and making your decision, you will be asked to answer some questions about your personal data, your illness, and a saliva sample and a nasopharyngeal swab sample will be taken; These samples will only be taken once.

3. Who participates?

All those people with suspected COVID-19 infection, who present mild signs and symptoms and who have agreed to participate in the study.

4. What procedures will you perform?

If you participate in the study, a saliva sample of approximately 1 mL will be taken from you, for which you will be asked to spit into a specific container that will be provided to you. Likewise, a swab sample will be taken from your nasal and oral cavity. These procedures will allow us to determine whether the test that has been developed is as effective in detecting the presence of the SARS-CoV-2 virus as the molecular test (RT-qPCR). Your samples will be analyzed at the Universidad Peruana Cayetano Heredia and at the ROE Laboratory. You will know your results as soon as they are available (maximum 72 hours), these will be communicated to you in a timely manner by the person in charge of the study.

5. What risks, discomforts or annoyances will the participants have?

Taking the saliva sample will not cause you any discomfort or discomfort; For its part, obtaining a nasopharyngeal sample with a swab could cause some discomfort at the nasal level and in the throat, as well as possible temporary tearing.

6. What benefits will you have if you participate?

You will not receive any type of financial compensation for your participation in this study, nor will you be charged for it. However, the results will allow a better decision in your diagnosis and with it your treating physician can establish the most appropriate treatment.

7. Will my identity be known?

Your sample and its results will be handled with the utmost confidentiality. Your name or any other information that could identify you will never be published.

8. Who can be asked for more information?

You can ask questions about anything that is not clear, now or in the future. If you wish to make further inquiries related to the

COMITE INSTITUCIONAL DE ETICA UNIVERSIDAD PERUANA CAYETANO HEREDIA
APROBADO
F. APROBACIÓN 20/01/21

Machine Translated by Google

study, you can contact the Principal Investigator of the study, Joaquín Abugattas Núñez del Prado, by phone: 994673230 or email: joaquin.abugattas.n@upch.pe

This consent has been reviewed and approved by the Social Security Health Research Ethics Committee. The task of this committee is to ensure that participants are protected from harm. If you want to know more about this committee, you can contact us directly through the contact information provided.

9. Who can make a complaint or annoyance about the study?

You can call directly the President of the Institutional Committee of the Instituto Nacional Cardiovascular INCOR - ESSALUD, Dr. Cecilia Cuevas De La Cruz by phone (01) 4111560 during office hours (08:00-16:30 hrs) or by mail email: comitedeeticaincor@gmail.com.

CONSENT CERTIFICATE

I have read or have had read to me the information contained on all pages of this document.
I had the opportunity to ask questions related to my participation that were resolved in a satisfactory and understandable manner. I give my voluntary consent to participate

PARTICIPANT'S NAME: ____________________

PARTICIPANT SIGNATURE: ____________________

DATE AND TIME: __________

If the participant cannot read and/or sign:
Volunteer's right thumbprint:

COMITÉ INSTITUCIONAL DE ÉTICA UNIVERSIDAD PERUANA CAYETANO HEREDIA CIE
APROBADO
F. APROBACIÓN 20/01/21

Machine Translated by Google

Annex 3. Consent for the use of your sample for further studies

Study: "Field validation of a diagnostic test for SARS-CoV-2 using CRISPR CAS in saliva samples in symptomatic subjects from Lima, 2021"

Research Institutions:
Peruvian University Cayetano Heredia, Social Health Security (EsSalud)

Principal investigator:
Joaquin Abugattas Nunez del Prado

I have been informed about the possibility of storing the saliva and swab samples that have been taken from me as part of the study "Field validation of a diagnostic test for SARS-CoV-2 using CRISPR-CAS in saliva samples in symptomatic subjects of Lima, 2021", which would be kept in the laboratories of the Social Security of Health, and of the Peruvian University Cayetano Heredia at -80º C for a period of 05 years or until it is exhausted, in order to carry out studies solely intended for the understanding of the disease COVID 19. It has been explained to me that the samples that are stored will not present information about my identity; Likewise, I have also been told that if I do not wish to keep my sample for future studies, it will not affect my participation in the study; and that if I accept that they be kept, I have the power to revoke said authorization at any time. It is important to mention that if these samples are used for further research, these new research protocols will require new approval by a research ethics committee prior to their execution.

Please mark with a cross to express your consent on the conservation of your sample for future studies.

I authorize my nasopharyngeal swab samples to be stored for 05 years for future use in other research. (After this period of time they will be deleted).

ÿ Yes ÿ No

I authorize to have my saliva samples stored for 05 years for future use in other research. (After this period of time they will be deleted). ÿ Yes ÿ No

I have read the information, or it has been read to me. I have had the opportunity to ask questions about it and my questions have been answered to my satisfaction.

I consent voluntarily and understand that I have the right to withdraw my consent without affecting the current research study or my medical care.

PARTICIPANT'S NAME: ______________________________

PARTICIPANT SIGNATURE: ______________________________

DATE AND TIME: ______________

If the participant cannot read and/or sign:
Volunteer's right thumbprint:

COMITÉ INSTITUCIONAL DE ÉTICA
UNIVERSIDAD PERUANA CAYETANO HEREDIA
CIE
APROBADO
F. APROBACIÓN 20/01/21

Anexo 2: Consentimiento Informado

Estudio: "Validación de campo de una prueba diagnóstica de SARS-CoV-2 mediante CRISPR-CAS en muestras de saliva en sujetos sintomáticos de Lima, 2021"

Instituciones de Investigación:
Universidad Peruana Cayetano Heredia
Seguro Social de Salud

Investigador Principal:
Joaquín Abugattas Núñez del Prado

Introducción: Su intervención en este estudio es completamente voluntaria. Usted puede decidir no participar y esta decisión no afectará su tratamiento por su médico.

1. **¿Por qué se está realizando este estudio?**
La Universidad Peruano Cayetano Heredia (UPCH) y el Seguro Social de Salud (EsSalud) están realizando una investigación para validar una nueva prueba desarrollada en el Perú para detectar el virus SARS-CoV-2 causante de la enfermedad COVID-19 mediante la evaluación de una muestra de saliva.

2. **¿Cómo va a ser su participación?**
Después de recibir la información del estudio y de tomar su decisión, se le pedirá que responda unas preguntas sobre datos de su persona, su enfermedad y se le tomará una muestra de saliva y otra de hisopado nasofaríngeo; solo se le tomará estas muestras en una única vez.

3. **¿Quién participa?**
Todas aquellas personas con sospecha de infección de COVID-19, que presentan signos y síntomas leves y que han aceptado participar en el estudio.

4. **¿Qué procedimientos le realizarán?**
Si participa en el estudio, se le tomará una muestra de saliva de aproximadamente 1 mL, para ello se le pedirá que escupa en un recipiente específico que se le brindará. Asimismo, se le tomará una muestra de hisopado de su cavidad nasal y bucal. Estos procedimientos nos permitirán si la prueba que se ha desarrollado es tan efectiva para detectar la presencia del virus SARS-CoV-2 como lo es la prueba molecular (RT-qPCR). Sus muestras serán analizadas en la Universidad Peruana Cayetano Heredia y en los Laboratorio ROE. Usted conocerá sus resultados en cuanto estos estén disponibles (máximo 72 horas), estos se le serán comunicados oportunamente por el responsable del estudio.

5. **¿Qué riesgos, incomodidades o molestias tendrán los participantes?**
La toma de muestra de saliva no le generará ninguna incomodidad o molestia; por su parte, la obtención de muestra nasofaríngea con hisopo podría generarle alguna incomodidad a nivel nasal y en la garganta, así como un posible temporal lagrimeo.

6. **¿Qué beneficios tendrá si participa?**
Usted no recibirá ningún tipo de compensación económica por su participación en este estudio, ni tampoco se le cobrará por ello. Sin embargo, los resultados permitirán una mejor decisión en su diagnóstico y con ello que su médico tratante pueda establecer el tratamiento más adecuado.

7. **¿Se va a saber mi identidad?**
Su muestra y sus resultados serán manejados con la mayor confidencialidad. Nunca se publicará su nombre ni ninguna otra información que pueda identificarlo.

8. **¿A quién se le puede pedir más información?**
Usted puede hacer preguntas acerca de cualquier aspecto que no esté claro, en este momento o en el futuro. Si Usted desea hacer consultas más adelante relacionadas al

COMITÉ INSTITUCIONAL DE ÉTICA UNIVERSIDAD PERUANA CAYETANO HEREDIA CIE
APROBADO
F. APROBACIÓN 20/01/21

estudio, puede contactar con la investigadora Principal del estudio, Joaquín Abugattas Núñez del Prado, al teléfono: 994673230 o al email: joaquin.abugattas.n@upch.pe

Este consentimiento ha sido revisado y aprobado por el Comité de Ética en Investigación del Seguro Social de Salud. La tarea de este comité es asegurar que los participantes estén protegidos de daños. Si Usted desea conocer más acerca de este comité puede contactarse directamente a través de los datos de contacto proporcionados.

9. **¿A quién se le puede presentar alguna queja o molestia sobre el estudio?**
   Puede usted llamar directamente con la presidente del Comité Institucional de Instituto Nacional Cardiovascular INCOR - ESSALUD, Dra. Cecilia Cuevas De La Cruz a través del teléfono (01) 4111560 en horario de oficina (08:00-16:30 hrs) o al correo electrónico: comitedeeticaincor@gmail.com.

CERTIFICADO DE CONSENTIMIENTO

He leído o me ha sido leída la información contenida en todas las páginas de este documento. Tuve la oportunidad de hacer preguntas relacionadas con mi participación que fueron resueltas de manera satisfactoria y entendible. Doy mi consentimiento voluntario para participar

NOMBRE DEL PARTICIPANTE: ____

FIRMA DEL PARTICIPANTE: ____

FECHA Y HORA: ____________________

Si el participante no puede leer y/o firmar:
Huella del pulgar derecho del voluntario:

COMITÉ INSTITUCIONAL DE ÉTICA UNIVERSIDAD PERUANA CAYETANO HEREDIA CIE
APROBADO
F. APROBACIÓN 20/01/21

Anexo 3. Consentimiento para el uso de su muestra para estudios posteriores

Estudio: "Validación de campo de una prueba diagnóstica de SARS-CoV-2 mediante CRISPR-CAS en muestras de saliva en sujetos sintomáticos de Lima, 2021"

Instituciones de Investigación:
Universidad Peruana Cayetano Heredia, Seguro Social de Salud (EsSalud)

Investigador Principal:
Joaquín Abugattas Núñez del Prado

Se me ha informado sobre la posibilidad de almacenar las muestras de saliva e hisopado que se me han tomado como parte del estudio "Validación de campo de una prueba diagnóstica de SARS-CoV-2 mediante CRISPR-CAS en muestras de saliva en sujetos sintomáticos de Lima, 2021", las cuales serían conservadas en los laboratorios del Seguro Social de Salud, y de la Universidad Peruana Cayetano Heredia a -80º C por un período de 05 años o hasta que se agote, con el fin de realizar estudios únicamente destinados a la comprensión de la enfermedad COVID-19. Se me ha explicado que las muestras que sean almacenadas no presentaran información sobre mi identidad; así también se me ha señalado que en caso no desee conservar mi muestra para posteriores estudios no afectará mi participación en el estudio; y que de aceptar que se conserven tengo la potestad de revocar dicha autorización en cualquier momento. Es importante mencionar que en caso se utilizarán estas muestras para posteriores investigaciones, estos nuevos protocolos de investigación necesitarán una nueva aprobación por un comité de ética en investigación previo a su ejecución.

Por favor marcar con un aspa para manifestar su consentimiento sobre la conservación de su muestra para posteriores estudios.

Autorizo a tener mis muestras de hisopado nasofaríngeo almacenadas por 05 años para un uso futuro en otras investigaciones. (Después de este periodo de tiempo se eliminarán).

☐ Si ☐ No

Autorizo a tener mis muestras de saliva almacenadas por 05 años para un uso futuro en otras investigaciones. (Después de este periodo de tiempo se eliminarán).

☐ Si ☐ No

He leído la información, o me la han leído. He tenido la oportunidad de hacer preguntas acerca de ello y mis preguntas han sido respondidas satisfactoriamente.

Consiento voluntariamente y entiendo que tengo el derecho de retirar mi consentimiento sin que esto afecte el estudio de investigación actual o mi atención médica.

NOMBRE DEL PARTICIPANTE: ____________________________________

FIRMA DEL PARTICIPANTE: ____________________________________

FECHA Y HORA: __________________

Si el participante no puede leer y/o firmar:
Huella del pulgar derecho del voluntario:

COMITÉ INSTITUCIONAL DE ÉTICA UNIVERSIDAD PERUANA CAYETANO HEREDIA DUICT CIE
APROBADO
F. APROBACIÓN 20/01/21
